# Supplementary material for: Barriers to accessing mental health services for women with perinatal mental illness: systematic review and meta-synthesis of qualitative studies in the UK
Source: BMJ Open. 2019 Jan 24;9(1):e024803. doi: 10.1136/bmjopen-2018-024803 (PMC6347898; doi:10.1136/bmjopen-2018-024803)
Supplement: Supplementary file 1 [file bmjopen-2018-024803supp001.pdf]

Supplementary File 1: Full Search Strategy for Medline via Ovid

1. exp Postnatal Care/
2. exp Perinatal Care/
3. exp Prenatal Care/
4. exp Pregnancy/
5. exp Pregnant Women/
6. exp Parturition/
7. (Perinatal or postnatal or prenatal or pregnan* or antenatal or maternal or postpartum or birth).mp.
8. **OR 1-7**
9. exp Mental Disorders/
10. exp Mental Health/
11. ((mental adj5 health) or (mental adj5 health adj2 problem*) or (mental adj5 illness*) or well-being or psychosis or schizophreni* or anxi* or depressi* or (obsessive compulsive adj5 disorder) or phobi* or (personality adj2 disorder*) or substance abuse).mp.
12. **OR 9-11**
13. exp Health Services Accessibility/ or exp "Delivery of Health Care"/
14. (barrier* or (help adj2 seek*) or (health adj2 seek*) or (support adj2 seek*) or access).mp.
15. **OR 13-14**
16. exp Qualitative Research/
17. exp "Attitude of Health Personnel"/ or exp Health Knowledge, Attitudes, Practice/
18. (Experience* or attitude* or perspective*).mp.
19. (qualitative Research or qualitative or phenomenograph* or grounded theory or ethnograph* or case stud* or interview* or focus group*).mp.
20. **OR 16-19**
21. **8 AND 12 AND 15 AND 20**
22. limit 21 to (english language and yr="2007 -Current")
