# Supplementary material for: Barriers to accessing mental health services for women with perinatal mental illness: systematic review and meta-synthesis of qualitative studies in the UK
Source: BMJ Open. 2019 Jan 24;9(1):e024803. doi: 10.1136/bmjopen-2018-024803 (PMC6347898; doi:10.1136/bmjopen-2018-024803)
Supplement: Supplementary file 2 [file bmjopen-2018-024803supp002.pdf]

Supplementary Table 1: Comprehensive summary of data extracted from papers for review

	Author /Year	Study Location	Study Aims	Data collection Method	n =	Study Population	Analytic Strategy and interpretative Theory	Key Emergent Themes Relating to Barriers in Care	CASP Score (/10)
1	Almond (2011)	Southern England	Understanding equity in provision of PND services	Observed discussions between HVs and postnatal women and interviews	46	HVs (n=16) managers (n=6) English women (n=12) Bangladeshi women (n=9), cohesion worker (n=2) and mental health practitioner (n=1).	Thematic content data analysis	-Unacceptability of PND screening<br>-Policy not implemented in practice<br>-Cultural and language barriers	8
2	Baldwin (2009)	Inner London	Assessment of South Asian mothers well-being by Specialist community public health nurses	Interviews	8	SCPHN working with South Asian mother	Content analysis	-Poor support networks<br>-Culturally specific barriers<br>-Domestic and emotional abuse	5
3	Boath (2013)	Stoke-on-Trent	Teenage mothers' experiences of PND and their care	In-depth interviews	15	First-time mothers aged 16-19 who scored >12 on EPDS	Thematic Framework Analysis	-Consequences of disclosure<br>-Poor continuity of care with preferred HCP<br>-Utilisation of family/friends as alternatives for PNMH services<br>-Poor education about perinatal mental health (PNMH) and mental health services (MHS)<br>-Logistical issues	9
4	Boddy (2017)	England	Men's experiences of partner's admission to MBU for first episode PP	In-depth interviews	7	Partners of women receiving inpatient treatment for first episode PP	Interpretive Phenomenological Analysis	-Normalising symptoms<br>- Poor PNMH education<br>-Failures in system to manage emergencies<br>-PNMH as a barrier	10
5	Brown (2009)	Coventry, Warwickshire	Improve understanding of PNMH service access	Focus-group discussions	68	Midwives (n=50) and midwifery students (n=18) working with PMIs	Thematic Framework Analysis	-Unacceptability of PND screening<br>-HCP reluctant to diagnose<br>-Midwives have poor training on PNMH<br>-Normalising symptoms<br>-Policy not implemented in practice<br>-Unclear referral pathways<br>-Delays being seem by specialist<br>-Failures in system to manage emergencies	4
6	Chew-Graham (2008)	Bristol, Manchester London	Determine GPs' and HVs' roles in management of PND	In-depth interviews	23	GPs (n=(n=19) and HVs (n=14) from RESPOND trial	Inductive thematic analysis approach	-HCPs' reluctant to diagnose<br>-Unacceptability of PND screening<br>-Normalising symptoms<br>-Poor education about PNMH and available treatments<br>-Poor continuity of care and communication<br>-Inappropriate HCP attitudes	8

7	Chew-Graham (2009)*	Bristol, Manchester London	GPs, HVs and women's views on disclosure of PND symptoms	In-depth interviews	28	Women from RESPOND trial scoring >11 on EPDS	Inductive thematic analysis approach	-PNMH diagnosis issues<br>-PNMH as a barrier<br>-Stigma and fear of medications<br>-fear of "bad mum" label<br>-poor opinion of HCPs<br>-Unclear roll of HV<br>-"easier not to ask"<br>-inadequate resources	8
8	Coates (2015)	South East England	PNMH symptoms in new mothers and experiences of assessment	interviews at home (n=15) or via telephone (n=2)	17	Women experiencing distress with baby <1year	Inductive thematic analysis approach<br>----------------------<br>*Critical realist approach*	-PNMH diagnosis issues<br>-Unacceptability of PND screening<br>-"it is actually alright to ask for help"<br>-Stigma of PNMH<br>-Inadequate resources<br>-Logistically issues	10
9	Coates (2014)	South East England	Experiences of postpartum emotional distress	interviews at home (n=15) or via telephone (n=2)	17	Women experiencing distress with baby <1year	Interpretive Phenomenological Analysis	-Fear of "bad mum" label<br>-Normalising symptoms<br>-Waiting for help to come<br>-Poor opinion of HCP<br>-Poor support networks	10
10	Cooke (2012)	UK**	Parents' perceptions of barriers to PNMH services	In-depth interviews	7	Parents (>18years) with psychological distress	Thematic Framework Analysis	-Fear of disclosure<br>-Inappropriate HCP attitudes<br>-PNMH as barrier	5
11	Edge (2007)	Northern England	Explain low PND service access among Black Caribbean women	In-depth interviews	12	Black Caribbean women, >18years, scoring >12 on EPDS	Grounded theory approach using constant comparison method	-Poor PNMH awareness<br>-Culturally specific barriers<br>-Fear of "bad mum" label<br>-Fear of medication side effects<br>-Fear of disclosure and stigma<br>-Inappropriate HCPs attitudes	8
12	Edge (2008)	North West England	Explain low PND service access among Black Caribbean women their absence from UK perinatal research	In-depth interviews	12	Black Caribbean women, >18years, scoring >12 on EPDS	Thematic Framework Analysis	-Culturally specific barriers<br>-Inappropriate HCP attitudes<br>-Stigma	10
13	Edge and MacKian (2010)	North West England	Black Caribbean women's accounts of help-seeking for PND	In-depth interviews	12	Black Caribbean women, >18years, scoring >12 on EPDS	Thematic Framework Analysis<br>------------------------<br>Draws on social model of depression and MacKian concepts of 'reflexive Communities'	-Culturally specific barriers<br>-Poor PNMH awareness	9

14	Edge (2010)	Northern England	HCPs views about PNMH care for BME women	In-depth interviews and Focus Group discussions	42	GPs (n=5), midwives (n=22), midwifery managers (n=5), hospital doctors (n=2), HVs (n=5), volunteers (n=3)	Thematic Framework Analysis	-Physical health over PNMH<br>-Policy not implemented<br>-Diagnostic difficulties<br>-Poor interdisciplinary communication<br>-Unclear referral pathways<br>-Lack of resources<br>-Language barriers	9
15	Edge (2011)	Northern England	Black Caribbean women's opinion of PND service access	In-depth interviews	42	Black Caribbean women >18years, with PND or knew someone who had PND	Thematic Framework Analysis	-Previous negative NHS experience<br>-Inadequate resources<br>-Poor support networks<br>-Culture barriers, poor awareness of PNMH	9
16	Evans (2017)	East Midlands	Women's experiences of anxiety in pregnancy and their views on the use of anxiety instruments in antenatal care	Focus group discussions	19	Woman >18 years old who had given birth in last nine months. Women had to have considered themselves as having anxiety during pregnancy	Template analysis	-Not able to share worries with others and feeling isolated<br>-Poor communication<br>-Unclear referral pathways<br>-Assessment tools as a tick box exercise	7
17	Gardner (2014)	Manchester	UK-based West African mothers experiences of PND	In-depth interviews	6	Black West African women scoring >10 on EPDS and using self-help parenting groups	Interpretive Phenomenological Analysis	-Poor PND awareness<br>-Poor support networks<br>-Lack of religious faith<br>-Logistical issues<br>-Stigma	8
18	Glover (2014)	Northern England	Women's experiences of PP and their causal explanations	In-depth interviews	7	Women accessing specialist psychiatric services due episode of PP <10years ago	Inductive thematic analysis<br>-------------------------<br>Draws on the stress-vulnerability model	-Normalising symptoms<br>-Poor advice from friends<br>-Poor education about PP<br>-Baby as a barrier<br>-Focus on physical health<br>-Stigma	9
19	Husain (2015)	Manchester Lancashire	Pilot trial of culturally-adapted PHP for British South Asian women with PND	In-depth interviews (Conducted in Urdu, transcribed in English)	17	British South Asian Women with PND from Bangladesh (n=1) Pakistan (n=14) India (n=2). All participants from ROSHNI-2 trial	Thematic Framework Analysis	-Domestic and emotional abuse<br>-Logistical issues<br>-Language and cultural barriers<br>-Lack of anonymity in therapy<br>-Anxiety about treatment	8
20	Jomeen (2013)	Northern England	HVs' experiences of psychological distress and PMI assessment	Focus Group Discussions	5	HVs attending an optional PNMH training day	Thematic Framework Analysis	-Lack of formal PNMH training<br>-Diagnostic issues<br>-Stigma<br>-Issues with interpreters<br>-Poor opinion of HCPs<br>-Issues with referral pathway<br>-Interdisciplinary communication	9

21	Lam (2012)	Manchester	Investigate Chinese women's postnatal experiences	In-depth interviews (conducted in Chinese and English)	8	UK-based, postnatal, Chinese women. EPDS from 2-17***	Grounded theory approach	-Poor information available<br>-Utilisation of family, friends and online forums as an alternative for PNMH services<br>-Language barrier<br>-Fear of family conflict<br>-Conflicting advice from professionals and family<br>-preoccupied with baby	9
22	McGookin (2017)	Manchester	Student Midwives awareness, knowledge and experience of antenatal anxiety	Semi-structured in-depth interviews (19-30 minutes)	7	Student Midwives (year 1-3)	Thematic analysis	-Fear of consequences of disclosure<br>-lack of education and awareness about mental health<br>-poor training<br>-inappropriate staff attitudes<br>-Lack of time and resources	7
23	McGrath (2013)	North West England	Understanding PP recovery	In-depth interviews conducted via phone (n=1) or face-to-face (n=11)	12	Women with any postnatal psychosis according to International classification of Disease- version 10 (ICD-10)	Grounded theory approach<br>---------------------<br>Draws on 'Recovery framework' and 'Biopsychosocial models of illness'	-PNMH as a barrier<br>-Fear of "bad mum" label<br>-Stigma	10
24	Millet (2018)	London	Women's and HCPs experience of access to *Improving Access to Psychological therapies* (IAPT) services during perinatal period	Interviews with service users (n=12) and therapists (n=14)	26	Women referred to mental health services who spoke English and had a baby aged 6-9 months at time of interview. Therapists working for IAPT.	Thematic Analysis	-Normalising symptoms<br>-Fear of "bad mum" label and consequences of disclosure<br>-IAPT not tailored to individual with PNMH conditions<br>-Non baby-friendly environment<br>-Lack of family support	9
25	Nicholls (2007)	Sussex	Experiences of postnatal-PTSD in couples relationship	In-depth interviews	12	Couples where either women (n=3), partner (n=1) or both (n=2) fulfilled DSM-IV criteria for postnatal-PTSD ****	Thematic Framework Analysis	-Difficulty of discussing PNMH<br>-Support networks<br>-Previous negative NHS experience	8
26	Patel (2013)	North West of England	PND illness beliefs	In-depth interviews	11	Women with PND or depression in pregnancy which continued after birth and scored >10 on EPDS	Grounded Theory<br>------------------------<br>Draws on 'Leventhal's self-regulation model of illness beliefs'	-Fear of "bad mum" label<br>-Normalising symptoms and unknown cause of symptoms<br>-Stigma<br>-PNMH as barrier	9
27	Phillips (2015)	UK	Student midwives awareness of PMIs	Focus group discussions (2 groups lasting 1 hour)	9	Student midwives within 6 months of completing degree.	Thematic Framework Analysis	-Poor resources<br>-"easier not to ask"<br>-Inadequate education about PNMH	8

28	Plunkett (2017)	North West England	Role of baby in PP recovery from	In-depth interviews phone (n=7) or face-to-face (n=5)	12	Women who had experienced PP. (checked on a "symptom checklist")	Thematic Framework Analysis<br>---------------------<br>*****	-Baby as motivating recovery factor<br>-Fear of disclosure	10
29	Radcliffe (2011)	Three UK hospital trusts	Provider experiences of services access and stigmatisation for substance-misusing women	In-depth interviews and participant observations	46	Community midwives, specialist midwives, postnatal ward staff, sonographers (n=22). Perinatal women accessing substance-misuse clinics (n=24)	Grounded Theory<br>---------------------<br>Uses a 'social constructionist approach'	-Poor diagnostic tools<br>-Stigma<br>-Logistical issues (eg. lack of diary to keep appointments)	4
30	Rothera (2008)	Trent, Leicestershire, Northampton, Rutland	To highlight issues with PNMH management	In-depth interviews and Focus Group discussions	39	HVs(n=4), Midwife (n=4), GP's (n=3), mental health practitioner (n=2), community psychiatric nurse (n=8), obstetrician (n=5), psychiatrists (n=9), managers (n=4)	Analytic induction and data reduction	-Failure to manage emergencies<br>-Policy not implemented<br>-Poor HCP PHM education and awareness<br>-Interdisciplinary communication	7
31	Rowan (2010)	Southern England	Exploration of policy recommendation implementation	In-depth interviews	8	Managers of maternity services, community midwives, community psychiatric nurses and psychiatrists	Thematic Framework Analysis	-Policy not implemented<br>-Issues with catchment areas<br>-Poor awareness about PNMH treatments	8
32	Slade (2010)	Trent	Women's experiences of HVs identifying and managing PND (part of PoNDER trial).	In-depth interviews	30	Women in PoNDER trial with 6-week PNDS score >18	template approach	-Poor support networks<br>-Fear of disclosure and stigma<br>-Unclear role of HV and poor training<br>-Poor continuity of care<br>-Inadequate resources<br>-Negative experiences of HCP	9
33	Wan (2008)	Manchester Liverpool	Explored providers' perspectives of support needs of mothers with schizophrenia	In-depth interviews	28	Midwives (n=15), obstetric consultant (n=1), psychiatrist (n=1), registered mental health nurses (n=7), manager (n=1) nursery nurse (n=3). Recruited from MBUs (n=9) or antenatal clinics (n=19).	Thematic Framework Analysis	-Fear of consequences<br>-Lack of midwifery training<br>-PNMH as barrier<br>-Interdisciplinary communication<br>-Lack of education about PNMH<br>-Continuity of care<br>-Support networks<br>-Lack of resources	10
34	Wittkowski (2011)	Greater Manchester	South Asian mothers' understanding of PND	In-depth interviews	10	South Asian mothers scoring >12 on EPDS	Grounded Theory approach	-Fear of upsetting others, losing job and stigma<br>-Cultural and language barriers<br>-Racial discrimination<br>-Poor PNMH awareness<br>-Poor social support networks<br>-Interdisciplinary communication	8

35	Wyatt (2015)	England	How women and their significant others make sense of PP experiences	In-depth interviews	7	Women with PP and their carers (partners (n=5), family members (n=1) friends (n=1))	Interpretive Phenomenological Analysis	-Poor social support networks<br>-Fear of "bad mum" label<br>-Poor knowledge of PNMH amongst carers<br>-Feeling guilty for feeling ill	9

Table 4.1 Key:

**Quotes from the Chew-Graham 2008 study population were also included in this paper*

***Paper quotes: "Culturally and socio-economically diverse, urban region of UK"*

****This review will only extract data from participants scoring >10 on EPDS*

*****This review will only extract data from the women within the study diagnosed with birth-related PTSD*

******Paper mentions there is limited theory and pre-existing research on this topic so recognises it may not be possible to map findings onto a formal theory*
