# Supplementary material for: Barriers to accessing mental health services for women with perinatal mental illness: systematic review and meta-synthesis of qualitative studies in the UK
Source: BMJ Open. 2019 Jan 24;9(1):e024803. doi: 10.1136/bmjopen-2018-024803 (PMC6347898; doi:10.1136/bmjopen-2018-024803)
Supplement: Supplementary file 3 [file bmjopen-2018-024803supp003.pdf]

Supplementary Table 2: Summary of CASP tool used for quality appraisal of Qualitative Data

Key: ✓= Yes; X= No; ?= Insufficient information to make assessment

Author/Year	Clear Aims	Appropriate methodology	Appropriate research design	Appropriate recruitment strategy	Appropriate data collection methods	Researcher-participant relationship considered	Ethical issues considered	Rigorous data analysis	Clear findings	Value of research	Overall Assessment
Almond (2011)	✓	✓	?	✓	✓	✓	✓	✓	✓	?	Adequate
Baldwin (2009)	✓	?	✓	✓	✓	?	✓	?	X	?	Weak
Boath (2013)	✓	✓	✓	✓	✓	?	✓	✓	✓	✓	Strong
Boddy (2017)	✓	✓	✓	✓	✓	✓	✓	✓	✓	✓	Strong
Brown (2009)	✓	✓	✓	?	?	?	?	?	✓	?	Weak
Chew-Graham (2008)	✓	✓	✓	✓	✓	?	?	✓	✓	✓	Adequate
Chew-Graham (2009)	✓	✓	✓	✓	✓	?	✓	✓	✓	?	Adequate
Coates (2015)	✓	✓	✓	✓	✓	✓	✓	✓	✓	✓	Strong
Coates (2014)	✓	✓	✓	✓	✓	✓	✓	✓	✓	✓	Strong
Cooke (2012)	X	✓	✓	?	?	✓	✓	?	✓	?	Weak
Edge (2007)	✓	✓	✓	✓	✓	?	✓	✓	?	✓	Adequate
Edge (2008)	✓	✓	✓	✓	✓	✓	✓	?	✓	✓	Strong
Edge and MacKian (2010)	✓	✓	✓	✓	✓	?	✓	✓	✓	✓	Strong
Edge (2010)	✓	✓	✓	✓	✓	?	✓	✓	✓	✓	Strong
Edge (2011)	✓	✓	✓	✓	✓	?	✓	✓	✓	✓	Strong
Evans (2017)	✓	✓	✓	✓	✓	?	✓	?	✓	?	Adequate
Gardner (2014)	✓	✓	✓	✓	✓	?	✓	?	✓	✓	Adequate
Glover (2014)	✓	✓	✓	✓	✓	?	✓	✓	✓	✓	Strong
Husain (2015)	✓	✓	✓	✓	✓	✓	✓	?	✓	?	Adequate
Jomeen (2013)	✓	✓	✓	?	✓	✓	✓	✓	✓	✓	Strong
Lam (2012)	✓	✓	✓	✓	✓	✓	?	✓	✓	✓	Strong
McGookin (2017)	✓	✓	✓	✓	✓	?	✓	?	✓	?	Adequate
McGrath (2013)	✓	✓	✓	✓	✓	✓	✓	✓	✓	✓	Strong
Millet (2018)	✓	✓	✓	?	✓	✓	✓	✓	✓	✓	Strong
Nicholls (2007)	✓	✓	✓	✓	?	?	✓	✓	✓	✓	Adequate
Patel (2013)	✓	✓	✓	?	✓	✓	✓	✓	✓	✓	Strong
Phillips (2015)	✓	?	✓	✓	✓	✓	✓	?	✓	✓	Adequate
Plunkett (2017)	✓	✓	✓	✓	✓	✓	✓	✓	✓	✓	Strong
Radcliffe (2011)	X	✓	?	?	✓	?	✓	✓	X	?	Weak
Rothera (2008)	✓	✓	✓	✓	✓	?	?	?	✓	✓	Adequate
Rowan (2010)	✓	✓	✓	✓	✓	?	✓	?	✓	✓	Adequate
Slade (2010)	✓	✓	✓	✓	✓	?	✓	✓	✓	✓	Strong
Wan(2008)	✓	✓	✓	✓	✓	✓	✓	✓	✓	✓	Strong
Wittkowski (2011)	X	✓	✓	?	✓	✓	✓	✓	✓	✓	Adequate
Wyatt (2015)	✓	✓	✓	?	✓	✓	✓	✓	✓	✓	Strong
