# Supplementary material for: Barriers to accessing mental health services for women with perinatal mental illness: systematic review and meta-synthesis of qualitative studies in the UK
Source: BMJ Open. 2019 Jan 24;9(1):e024803. doi: 10.1136/bmjopen-2018-024803 (PMC6347898; doi:10.1136/bmjopen-2018-024803)
Supplement: Author's manuscript [file bmjopen-2018-024803.draft_revisions.pdf]

BMJ Open

BMJ Open is committed to open peer review. As part of this commitment we make the peer review history of every article we publish publicly available.

When an article is published we post the peer reviewers' comments and the authors' responses online. We also post the versions of the paper that were used during peer review. These are the versions that the peer review comments apply to.

The versions of the paper that follow are the versions that were submitted during the peer review process. They are not the versions of record or the final published versions. They should not be cited or distributed as the published version of this manuscript.

BMJ Open is an open access journal and the full, final, typeset and author-corrected version of record of the manuscript is available on our site with no access controls, subscription charges or pay-per-view fees (http://bmjopen.bmj.com).

If you have any questions on BMJ Open's open peer review process please email editorial.bmjopen@bmj.com

BMJ Open

Barriers to Accessing Mental Health Services for Women with Perinatal Mental Illness: Systematic Review and Meta-Synthesis of Qualitative Studies in the UK

Journal:	*BMJ Open*
Manuscript ID	bmjopen-2018-024803
Article Type:	Research
Date Submitted by the Author:	27-Jun-2018

Complete List of Authors:	Megan Sambrook Smith, Megan; King's College London, Institute of Psychiatry, Psychology and Neuroscience, Global Mental Health<br>Lawrence, Vanessa; Kings College, Qualitative Social Sciences<br>Sadler, Euan; Kings College London, Centre for Implementation Science, Health Service & Population Research Department,<br>Easter, Abigail; King's College London, Institute of Psychiatry, Psychology and Neuroscience, Centre for Implementation Science, Health Service & Population Research Department,

Keywords:	Barriers, Systematic Review, MENTAL HEALTH, Pregnancy, QUALITATIVE RESEARCH

SCHOLARONE™
Manuscripts

For peer review only

Barriers to Accessing Mental Health Services for Women with Perinatal Mental Illness: Systematic Review and Meta-Synthesis of Qualitative Studies in the UK

AUTHORS:

Sambrook Smith, M., MSc[1], Lawrence, V., PhD[2] Sadler, S., PhD[3], Easter, A. PhD[3]

1. Global Mental Health, Institute of Psychiatry, Psychology & Neuroscience, King's College London, 16 De Crespigny Park, London SE5 8AF
2. Health Service and Population Research Department, Institute of Psychiatry, Psychology & Neuroscience, King's College London, PO26, The David Goldberg Centre, De Crespigny Park, London, SE5 8AF
3. Centre for Implementation Science, Health Service & Population Research Department, Institute of Psychiatry, Psychology & Neuroscience, King's College London, De Crespigny Park, London, SE5 8AF

CORRESPONDING AUTHOR

Dr Abigail Easter

Email: abigail.easter@kcl.ac.uk

Phone: 020 7848 5548

WORD COUNT: 3,999

For peer review only

ABSTRACT

Objective: Lack of access to mental health services during the perinatal period is a significant public health concern in the UK. Barriers to accessing services may occur at multiple points in the care pathway. However, no previous reviews have investigated multi-level system barriers or how they might interact to prevent women from accessing services. This review examines women, their family members' and healthcare providers' perspectives of barriers to accessing mental health services for women with perinatal mental illness in the UK.

Design: A systematic review and meta-synthesis of qualitative studies.

Data Sources: Qualitative studies, published between January 2007 and June 2017, were identified in MEDLINE, PsychINFO, EMBASE, and CINAHL electronic databases, hand-searching of reference lists and citation-tracking of included studies. Quality assessment was conducted using the Critical Appraisal Skills Programme for qualitative studies.

Results: Of 8,054 papers identified, 32 studies met the inclusion criteria. Reporting of emergent themes were informed by an existing multi-level conceptual model. Barriers to accessing mental health services for women with perinatal mental illness were identified at four levels: Individual (e.g. stigma, poor awareness), organisational (e.g. resource inadequacies, service fragmentation), socio-cultural (e.g. language/cultural barriers), and structural (e.g. unclear policy) levels.

Conclusions: Complex, interlinking, multi-level barriers to accessing mental health services for women with perinatal mental illness exist. To improve access to mental healthcare for women with perinatal mental illness multi-level strategies are recommended which address individual, organisational, sociocultural, and structural level barriers at different stages of the care pathway.

Key Words: *barriers; mental health; pregnancy; qualitative studies; systematic review*

For peer review only

STRENGTHS AND LIMITATIONS OF THIS STUDY

- This study provides a comprehensive and robust systematic review of barriers to mental healthcare for women with perinatal mental illness, a key public health issue.
- Robust procedures for systematic reviewing and quality assessment were adopted, in line with PRISMA reporting guidelines.
- Unidentified barriers, specifically those at structural and organisational levels, may remain due to limited and high quality research in these areas.
- Due to the wide variability in the context of delivery of perinatal mental health care globally, this review only included studies conducted with the UK. The findings may therefore be less applicable to other healthcare settings.

For peer review only

INTRODUCTION

Approximately 10-20% of women experience mental illness during pregnancy or in the first postpartum year (perinatal period)[1-4]. Perinatal mental illnesses (PMI) are associated with increased morbidity and are a leading cause of maternal death in high-income countries[5, 6]. PMI may also adversely affect psycho-social development of offspring[7], and are associated with significant long-term socio-economic costs[1]. Timely identification and treatment of PMI by trained healthcare professionals (HCPs) is paramount.

The 'Five Year Forward View for Mental Health' aims to transform mental health services (MHS) in the UK, and identifies the need to improve perinatal mental health (PNMH) as a strategic priority for the National Health Service (NHS)[8]. A key recommendation is "by 2020/21, NHS England should support at least 30,000 more women each year to access evidence-based specialist mental healthcare during the perinatal period"[8]. However, in the UK an estimated 60% of women have no access to PNMH services[2] and 38% of women wait over a month to be referred[9]. Inadequacies in community mental health services, shortages of health visitors (HVs), and midwives and lengthy waiting lists for psychosocial therapies further limit access to MHS for women with PMI[8].

Barriers to care extend beyond inadequate resources[10]. One survey reported that 30% of women withheld negative feelings from HCPs often due to fear of their baby being taken away[2]. Previous reviews highlight that lack of mother-centred antenatal care, stigmatising attitudes towards mental health, and insufficient knowledge among HCPs about PMI contributed to help-seeking delays[11, 12]. There is growing evidence suggesting reasons for difficulties accessing MHS are more complex[13], potentially occur at multiple time points along the care pathway[10] and are compounded by socio-cultural and economic issues[14, 15]. However, no previous review has synthesised evidence on different stakeholder views of where these barriers exist or how they interact to hinder access to MHS during the perinatal period. Identifying where barriers exist is imperative to developing a comprehensive understanding of how to improve access to PNMH care.

This systematic review and meta-synthesis of qualitative studies in the UK examines barriers to accessing MHS for women with PMI from the perspective of women themselves, their family members and HCPs, and provides evidence to support the implementation of the Five Year Forward View Plan.

For peer review only

MATERIALS AND METHODS

We conducted a systematic review and meta-synthesis of qualitative studies.

Search strategy and selection criteria

This systematic review adhered to the PRISMA checklist for reporting findings of systematic reviews and followed a predetermined published protocol registered on the International Prospective Register of Systematic Reviews PROSPERO (ID number CRD42017060389); available at: https://www.crd.york.ac.uk/PROSPERO/display_record.php?RecordID=60389)

The first author (MSS) initially searched *Ovid MEDLINE(R)*, *PsychINFO*, *EMBASE,* and *CINAHL* electronic databases between January 2007 to June 2017 using the following combination of keywords and MeSH terms: (“Perinatal” OR “Pregnancy” OR “Birth”) *AND* (“Mental Health” OR “Mental Disorder”) *AND* (“Health Service Accessibility OR “Delivery of Health Care”) AND (“Qualitative Research” OR “Attitudes of Health Personnel” OR “Health Knowledge, Attitudes, Practice”). MSS then hand-searched reference lists of included studies and used citation-tracking of these studies in Google Scholar to identify further relevant papers.

We included qualitative studies examining womens’, families’ and HCPs’ perspectives of barriers to accessing MHS for women with mental illness during the perinatal period, published in peer reviewed English language journals. We defined the perinatal period as any time from conception to the first year postnatal. We excluded studies with purely quantitative data or those not conducted in the UK to ensure findings related directly to “Five Year Forward” implementation.

All papers returned by searches were imported into Endnote (version X7·7·1) and duplicates removed. The first author conducted initial screening and study selection, then two independent reviewers assessed a random 10% sample (n=19) of full text search papers for eligibility (agreement measured using Cohen’s Kappa). Abstracts and titles of each paper were then read and full texts retrieved for studies deemed potentially relevant. Two authors (MSS, AE) discussed studies where inclusion was not clear.

Quality appraisal

The quality of all included studies was assessed using the Critical Appraisal Skills Programme (CASP) checklist for qualitative studies, which provides a framework for assessing the quality and rigour of selected studies[16]. CASP scores, ranged from 0-10, were used to categorise studies as either ‘strong’ (score 10/10; no methodological issues), ‘adequate’ (score >6/10; no major methodological issues), or ‘weak’ (score <4/10; major methodological issues) quality. Quality assessment was carried

For peer review only

out by the first author and then 20% (n=6) by two additional reviewers (ES, AE). Agreement was calculated using Cohen's Kappa and disagreements were resolved by discussion between MSS and AE. Studies deemed poor quality were not excluded from the review.

Data Extraction and synthesis approach

A meta-synthesis approach was used to synthesise findings from qualitative studies[17]. The approach was chosen to improve understanding and conceptual development greater than that attained from the individual studies alone[17]. We constructed data extraction tables to record key characteristics and summary findings from included studies (table 1: supplementary document). A constant comparison method approach was used to identify emerging themes and related subthemes, including discordant themes, looking for similarities and differences in stakeholder perspectives across all papers. These were then graphically displayed as a 'conceptual map' to visually display themes and explore relationships between themes and related subthemes[18]. All authors met regularly to discuss the emerging themes.

A theoretical multi-level conceptual framework based on the "delivery systems" model (figure 1)[19, 20] was subsequently used to help organise, report and interpret meta-synthesis findings. This adapted model, based on Ferlie and Shortell's "Framework for change"[20] in combination with Reid's "delivery system"[19], was created through discussion between two authors (MSS and ES) after reviewing included papers. This was to allow for specific individual, organisational, socio-cultural and structural level factors (e.g. policy and politico-economic factors) to be drawn out of the analysis and provided a theory-driven approach.

Figure 1 here

PATIENT AND PUBLIC INVOLVEMENT

The development of the research questions for this study was directly informed by the NHS 'Five Year Forward View for Mental Health' [8]. The priorities laid out in the Five Year Forward View were established by an independent Mental Health Taskforce, which brought together health and care leaders, service users and experts in the field. The findings will be disseminated widely to service user groups and voluntary organisations.

For peer review only

RESULTS

In total, we identified 9,480 articles, of which 27 qualitative studies met the eligibility criteria. A further five papers were identified through citation tracking. Therefore a total of 32 papers, reporting on 29 studies, were included for review (figure 2).

Table 1 (supplementary document) provides a summary table of study characteristics of included qualitative studies. Postnatal Depression (PND; n=13) and poor reported mental well-being (n=10) were the most commonly studied PMIs. Data collection was mostly via semi-structured interviews (n=28) with two studies using non-participant observations and six studies utilising focus groups. In 19 papers, the study population was women with PMI and 11 papers focused on HCPs working with these women, with four studies including family and friends as research participants.

Most studies were deemed either high (n=14) or moderate quality (n=14). Lower CASP scores were associated with poor reflexivity (e.g. researchers not considering how their own personal values affected the data collection) and non-rigorous data analysis methods. A low level of agreement in a sample of included studies between reviewers and the original CASP score given by MSS was seen (reviewer 1 (K)=0·2; reviewer 2 (K)=0). This was partly due to poor reporting of information resulting in difficulties assessing the true methodological quality of the studies. Considering these discrepancies, a discussion between MSS, AE and ES took place to reach a consensus on study quality of included studies.

Figure 2 here

Meta-synthesis of findings: Multi-level barriers to Mental Health Services

Barriers to accessing MHS for women with PMI related to a wide range of complex factors. Drawing on Reid's delivery system model[19], such factors operated on multiple-levels: individual (knowledge, attitudes and individual characteristics of women, their families and HCPs), organisational (organisational characteristics, service access, and inadequacy of resources), socio-cultural (family support, wider social support networks, and cultural attitudes), and structural (unclear policy) levels.

Individual level factors

Lack of knowledge about Perinatal Mental Health

Poor PMI awareness and knowledge among HCPs and women was cited in 13 studies as a barrier to accessing appropriate care[22, 26-37] . Unfamiliarity with the concept of PNMH and the signs and

For peer review only - http://bmjopen.bmj.com/site/about/guidelines.xhtml

symptoms of mental illness, as well as a perceived lack of open discussion between HCPs and family members were reported as common issues for women[22, 26, 27, 29-33, 36, 37]. One woman said:

> "I didn't really know the meaning of it [postpartum depression] …I could have detected it earlier if someone had explained to me what your first symptoms were, but nobody told me." (Teenage mother with PND)[27]

HCPs similarly reported poor knowledge of PMIs in a number of studies[28, 34, 35] which was often attributed to inadequate training opportunities[10, 23-25, 34, 35, 38]. One student midwife commented that "mental health is very challenging; we are not trained to give mental healthcare"[24]. Student midwives also highlighted gaps in the training curriculum as "there was only one lecture on mental health [and] no formal training"[10, 24].

Family members and friends of women with PMI played an important role in detecting signs and symptoms of illness. However, several studies found that family and friends could also hinder women from disclosing a mental illness to HCPs, often due to perceived stigma, leading to delays in seeking professional support [22, 26, 27, 32, 33, 36, 37, 39-41]. Family members also described feeling unable to recognise deteriorating signs and symptoms and therefore were unable to provide effective support[28]. Normalising symptoms of mental illness due to pregnancy and motherhood was highlighted in several studies as a way of explaining changes in maternal behaviour[10, 26, 28, 32, 41, 42]. Women with PMI commonly attributed symptoms (e.g. low mood and self-esteem) to tiredness or hormones, whereas partners and HCPs tended to dismiss such symptoms as part of the normal pregnancy experience. For example, one male spouse of a women with postpartum psychosis commented:

> "I… didn't really see the more acute signs because A. I'm not experienced in them and B. I knew there was something up but I put it down to her being absolutely over exhausted."[26]

Insufficient knowledge among HCPs about care pathways for women with PMI was reported in ten studies[22, 26-30, 34, 43-45]. In three of these studies, midwives, and HVs voiced a perceived lack of confidence, knowledge and skills to refer women to appropriate services[10, 21, 23] and obstetricians in one of the studies spoke about not knowing what local services were currently available[10], resulting in perceived delays in accessing services especially in complex cases[23] and emergencies[10, 25, 26].

Negative attitudes towards mental illness

For peer review only

Stigma, guilt, and shame associated with being given a PMI diagnosis and treatment was reported by women in 11 papers[23, 29, 31, 32, 38, 40, 42, 43, 46-48]. Sometimes women expressed feeling guilty about being ill at a time when happiness was expected[37, 42, 47]. Commonly concerns centred around negative consequences and stigma of disclosure, such as being labelled a 'bad mum'[29, 37, 40-42, 47], not wanting to upset other family members[33, 37], or not fulfilling perceived social expectations of motherhood[27, 29, 33, 35, 36, 38, 44, 49]. Women from minority groups particularly felt they were at risk of stigmatising attitudes from HCPs and the public due to cultural differences in social expectations[22, 27, 29, 36, 46]. For example, one Pakistani woman with PND said:

> "There is a huge stigma of being mentally ill in the public, but for us Asians there is a double disadvantage. I really fear that work will find out." [36]

Similarly, HCPs were sometimes reluctant to formally recognise symptoms related to PND because they did not want to impose labels on women[10, 28]. This was emphasised in one study among midwives who reported feeling uncomfortable about recording such concerns in women's medical notes which family members potentially had access to[24]. Furthermore, HCPs in six studies reported that women had refused treatment because of concerns around taking psychotropic medications, including the perceived stigma and feelings of failure as a good mother, fear of harm to their babies, and fears of dependence on medications and associated side effects[22, 23, 28, 29, 40, 45].

Organisational level factors

Inadequate resources

Inadequate resources in terms of staff shortages and limited service provision were reported by HCPs as key organisational barriers to providing effective services for women with PMI in a number of studies[21, 22, 24, 35, 38, 40, 43]. Midwives spoke about not having sufficient time to build rapport with women with PMI and some were "criticised as slow" by other HCPs if they were perceived to take more time[24]. In one study, a student midwife felt even if "information and knowledge can be there, there is no time" to provide support for women to access PNMH services, which shaped a sense of frustration[24].

Fragmented services: role clarity and conflict

For peer review only

Perceptions of poor continuity of care and not knowing which HVs to contact were reasons for non-disclosure among women with PMI in five studies[10, 27, 28, 35, 38]. Some HCPs described how a perceived lack of specialist services and long waiting lists adversely affected access to appropriate care[10, 25]. In one further study, variations in service organisation across different NHS Trusts in the UK were viewed by a range of HCPs to cause particular challenges in the healthcare system, and were perceived to compromise the creation of a “completely secure safety net” of care[34]. For example, one GP commented:

> “We have terrible trouble with HVs… because the HVs are now sectorised, we have to liaise with about 12 different HVs. It is just a nightmare! Deeply unsatisfactory! It's not the HVs' fault — it's the system.”[21]

Perceptions of fragmented services among HCPs were considered to cause problems with inter-disciplinary communication between professional groups, which hindered access of care for women with PMI[21, 23, 25, 28, 35, 36]. Communication was seen as particularly poor between primary care staff and mental health services[25, 28, 36, 40], in emergency situations[10, 26, 40] and during the handover of care from midwives to HVs[23, 35]. This left HVs in one study feeling frustrated and unsupported by other colleagues[23]. HVs in two further studies emphasised how fragmented services created confusion about the HV’s role within the referral pathway[38, 40]. Similarly, women were also confused about the HV role in supporting them to access appropriate care[38, 40], particularly in terms of liaising with social care providers. Women in a further study voiced uncertainty regarding knowing who was the most appropriate HCP to approach to access PND services[36]. For example, one woman with PND said:

> “My GP says go the HV and HV says go to GP. I don’t know what to do, I need help, don’t know where to go, or who to turn to”[36]

Socio-cultural level factors

Language barriers

Language as a barrier to accessing MHS and care was similarly reported by both mothers and HCPs in a third of included studies[21, 22, 29, 30, 33, 36, 39, 45, 46, 50]. Women from minority ethnic backgrounds felt they encountered significant barriers when requesting translators[33, 50]. For example, one Chinese woman said:

> “When the midwife visits, I can only speak the sentences about requesting a translator … They said that this kind of service is limited … that is what is difficult being Chinese – language barrier.”[33]

For peer review only

In one study midwives and HVs seemed to underestimate the importance of translators for such women or were frustrated at the extra work required to arrange such services[50]. This sometimes resulted in the overreliance on partners of women with PMI for translation which resulted in inaccuracies and ambiguity with exactly what the women wished to communication[50]. For example, one HV commented:

> "Because sometimes they say loads and then they come back saying, 'She said no'. I know that they've probably done it in shorthand." (HV)[50]

Differences in Cultural Values

The relationship between cultural attitudes, access to MHS and associated challenges this raised for women with PMI was an emerging theme in several studies[22, 29, 30, 33, 36, 39, 45, 46, 50]. The main barriers to accessing appropriate care for women from Black minority ethnic (BME) groups included dismissing mental health as a "something the doctors made up"[29], being unable to disclose feelings due to differences in ethnic backgrounds of HCPs[22, 29, 30, 46], and not receiving perceived culturally appropriate support (e.g. no available female doctors)[36]. With regards to the need to access specialist services, some women from minority ethnic groups in three studies spoke about the importance of the cultural competency of HCPs to promote and encourage help-seeking [29, 35, 50]. For example, one woman felt that she was met with culturally insensitive attitudes from her consultant:

> "I went to see the consultant about my hypertension a couple of weeks ago…and when I told him [about HV's 'diagnosis'], he said, 'you haven't got postnatal depression. You're too cheerful and bright and laughing" (BME woman with PND)[46]

Structural Level Factors

Unclear Policy around appropriate and acceptable use of assessment tools

A key theme among HCPs was the need for clearer policies to be implemented to address potential barriers to accessing MHS's for women with PMI. Polices discussed in various papers centred on the recommended use of appropriate assessment tools for diagnosis of PMI[10, 21, 23, 25, 28, 34, 43, 48, 50] and pathways of care[10, 21, 23]. HCPs frequently expressed negativity towards the use of existing assessment tools (such as the Edinburgh Postnatal Depression Scale (EPDS))[10, 21, 23, 25, 28, 34, 43, 48, 50]. Midwives, HVs and GPs agreed that such screening tools were currently

For peer review only

unsatisfactory[21, 23], and inconsistent usage was perceived to result in many women being missed in the system[43].

In contrast, women with PMI in three studies found the process of assessment therapeutic as they felt their symptoms were being taken seriously and had received formal recognition from professionals that they were unwell[38, 44, 50]. However, poor implementation of assessment tools by HCPs shaped negative perceptions among women in several studies of the care received. These included feeling that assessments were tick box exercises[43], conducted at inappropriate times[38, 43], and that findings sometimes did not reflect their experiences of PMI[43]. Lack of resources, treatment options, and poor knowledge of referral pathways also led to perceived ethical concerns amongst one HV who said:

"In an ideal world we'd want to pick them up and then offer them more support, but we can't do that. So there's almost this ethical dilemma of well is there any point in identifying them if you can't do anything with them other than send them to the GP for antidepressants, which isn't good, you know?" (HV)[28]

DISCUSSION

This review has identified multi-level barriers to accessing MHS for women with PMI in the UK during the perinatal period. In summary, we found that negative attitudes towards diagnosis and treatment of PMIs resulted in women avoiding help-seeking and reinforced feelings of stigma and guilt. Lack of PNMH knowledge among HCPs, women and their families led to poor recognition of symptoms, delayed referrals, and confusion over the role of the HV. Organisational level factors such as inadequate resources, fragmentation of services, and poor interdisciplinary communication compounded these individual level issues. Structural factors (especially poor policy implementation) and socio-cultural factors (e.g. language barriers) also caused significant barriers to accessing services for this group of women.

Based on the findings from this review, we propose a conceptual model to explain where these barriers fit within the care pathway for perinatal woman requiring MHS (figure 3).

Figure 3 here

The first stage of the care pathway involves identification of high-risk women and provision of general PNMH information to all pregnant women. We found that a key barrier to implementing this is poor general knowledge and education about MHS among women with PMI, their families, and HCPs[22, 26-37], especially among teenage[27], BME[21, 22, 29-31, 46] and South-Asian mothers[36, 39, 45]. Evidence has shown that midwives, who are best placed to discuss perinatal mental health

For peer review only - http://bmjopen.bmj.com/site/about/guidelines.xhtml

risk with pregnant women, receive inadequate PNMH education and training[51,52] with a high proportion reporting receiving no mental health training at all[51]. “Stepping Forward to 2020”[53], which outlines methods to achieve the “Five Year Forward View For Mental Health”[8], recommends development and implementation of a competency framework for staff. Our review supports this proposal, however there is also evidence from our review that women and their families had poor PNMH knowledge, highlighting the need for broader approaches to improve knowledge in these groups.

To provide appropriate support HCPs need to correctly identify common PNMH symptoms in distressed women and acknowledge symptoms in mothers who are not actively seeking help via routine mental health assessments (stage 2). Barriers to implementing this identified in this review include difficulties women have in differentiating between PNMH symptoms and “normal” pregnancy experiences, with some women lacking the insight and capacity to recognise they were unwell, especially those with PP[26, 32] and severe PND[28, 42]). Family members also play an important role in recognising symptoms of PMI[22, 26, 27, 32, 33, 36, 37, 39-41], however this was especially difficult for women from minority ethnic backgrounds, for example, those from Chinese[33], BME[29, 30, 46] and for South Asian women[36, 39, 45], with such subgroups largely not familiar with PNMH and presenting symptoms. High quality and culturally sensitive information about PNMH needs to be provided to each woman to highlight differences between perceived normal pregnancy changes and PNMH symptoms. Information needs to also include red flag signs, information for concerned family members, HCP contact information, and emergency protocols. Such resources should be available in multiple languages and adapted for cultural relevance.

Most studies in this review focused on factors influencing help-seeking among women with PMI (stage 3). Women often received conflicting advice about who best to approach in the system due to poor interdisciplinary communication between HCPs about their specific roles in the management of women with PMI[21, 23, 25, 28, 35, 36]. The “Stepping Forward” report proposes to create new job roles within PNMH care (e.g. psychological well-being practitioner) to address this issue[53]. However, to improve access to services there is also a need for clearer role clarification and understanding of referral pathways.

This review found that barriers preventing diagnosis (stage 4) of PMI among women mostly related to two factors: (1) issues with screening and diagnostic tools; and (2) HCPs reluctance to label mental health conditions due to fear of stigmatisation of the woman with PMI. Both factors, coupled with wider structural and organisational level barriers (e.g. limited resources) shaped complex ethical issues related to the diagnosis and routine screening of PMI for this group of women. The final stage

For peer review only

of the care pathway relates to receiving appropriate treatment for women with PMI. Increasing the number of community PNMH services and Mother and Baby Units will provide much needed additional resources[54], however it is clear from this review that for women to access these services implementation strategies that address barriers at earlier stages of the care pathway are crucial.

We acknowledge several possible limitations of our review. One possible limitation is that unidentified barriers, specifically those at structural and organisational levels, may remain due to limited research in these areas. Secondly, although most studies within the review were deemed of ‘strong’ or ‘adequate’ quality, there were gaps in reporting especially in terms of under-reporting of possible researcher bias during data collection and analysis. Small sample sizes in some of the included studies were another issue in terms of drawing out wider implications. However, the meta-synthesis approach we used enabled the pooling of emerging themes and related sub-themes, thus enhancing the robustness and credibility of the results from this review.

In conclusion, this systematic review and meta-synthesis of qualitative studies found multi-level barriers to accessing MHS for women with PMI in the UK. To make tangible and sustainable improvements to expand access to care for this group of patients, we advocate changes need to be implemented at several stages within the proposed care pathway, with specific attention given to targeting key barriers to accessing MHS for women with PMI. Furthermore, in increasing the number of specialist PNMH services and staff it is also vital that strategies are used to reduce individual, organisational, socio-cultural, and structural level barriers that women with PMI are facing in accessing MHS services in the UK. It will not be until these barriers are addressed that the targets outlined in the “Five Year Forward View for Mental Health” can be optimally met.

For peer review only

AUTHORS CONTRIBUTIONS

AE was responsible for the original conception and design of the work, with significant contributions from MSS, ES and VL. MSS was primarily responsible for conducting the review and data analysis, with quality appraisal checks on a sample of studies conducted by AE and ES. All authors made significant contributions to interpretation of the study findings. MSS produced the initial manuscript draft and further redrafts were critically revised and approved by all authors.

ACKNOWLEDGEMENTS

The research was supported by the National Institute for Health Research (NIHR) Collaboration for Leadership in Applied Health Research and Care (CLAHRC) South London at King's College Hospital NHS Foundation Trust, and the NIHR Biomedical Research Centre, Guy's and St Thomas' NHS Foundation Trust and King's College London, UK. The views expressed are those of the authors and not necessarily those of the NHS, the NIHR or the Department of Health.

FUNDING

AE and ES are funded through individual King's Improvement Science Fellowship awards. King's Improvement Science is part of the NIHR CLAHRC South London and comprises a specialist team of improvement scientists and senior researchers based at King's College London. Its work is funded by King's Health Partners (Guy's and St Thomas' NHS Foundation Trust, King's College Hospital NHS Foundation Trust, King's College London and South London and Maudsley NHS Foundation Trust), Guy's and St Thomas' Charity, the Maudsley Charity and the Health Foundation.

DECLARATION OF INTEREST

None

DATA SHARING STATEMENT

This study is a systematic review, all data included within the present study has been previously published and in the public domain.

For peer review only

References

1. Leight KL, Fitelson EM, Weston CA, Wisner KL: Childbirth and mental disorders. *Int Rev Psychiatry* 2010, 22(5):453-471.

2. Costs of perinatal mental health problems Centre for Mental Health: Personal Social Services Research Unit. [accessed 24/05/18]. Available at: [http://eprints.lse.ac.uk/59885/1/__lse.ac.uk_storage_LIBRARY_Secondary_libfile_shared_repository_Content_Bauer%2C%20M_Bauer_Costs_perinatal_%20mental_2014_Bauer_Costs_perinatal_mental_2014_author.pdf.]

3. Howard LM, Molyneaux E, Dennis CL, Rochat T, Stein A, Milgrom J: Non-psychotic mental disorders in the perinatal period. *Lancet* 2014, 384(9956):1775-1788.

4. O'Hara MW, Wisner KL: Perinatal mental illness: definition, description and aetiology. *Best Pract Res Clin Obstet Gynaecol* 2014, 28(1):3-12.

5. Wilkinson H: Saving mothers' lives. Reviewing maternal deaths to make motherhood safer: 2006-2008. *BJOG* 2011, 118(11):1402-1403; discussion 1403-1404.

6. Saving Lives, Improving Mothers' Care Lessons learned to inform future maternity care from the UK and Ireland Confidential Enquiries into Maternal Deaths and Morbidity 2009-2012. [accessed 24/05/18]. Available at: [https://www.npeu.ox.ac.uk/downloads/files/mbrrace-uk/reports/Saving%20Lives%20Improving%20Mothers%20Care%20report%202014%20Full.pdf.]

7. Stein A, Pearson RM, Goodman SH, Rapa E, Rahman A, McCallum M, Howard LM, Pariante CM: Effects of perinatal mental disorders on the fetus and child. *Lancet* 2014, 384(9956):1800-1819.

8. The five year forward for Mental Health: A report from the independent Mental Health Taskforce to the NHS in England February 2016. : Mental Health Taskforce strategy [https://www.england.nhs.uk/wp-content/uploads/2016/02/Mental-Health-Taskforce-FYFV-final.pdf]

9. Maternal Mental Health –Women's Voices. [accessed 24/05/18]. Available at: [https://www.rcog.org.uk/globalassets/documents/patients/information/maternalmental-healthwomens-voices.pdf.]

10. Brown M, Mills N, McCalmont C, Lees S: Perinatal mental health services: the problems in identifying and addressing mental health issues in pregnant women. *Primary Health Care* 2009, 19(8):36-39.

11. Dennis CL, Chung-Lee L: Postpartum depression help-seeking barriers and maternal treatment preferences: a qualitative systematic review. *Birth* 2006, 33(4):323-331.

12. Megnin-Viggars O, Symington I, Howard LM, Pilling S: Experience of care for mental health problems in the antenatal or postnatal period for women in the UK: a systematic review and meta-synthesis of qualitative research. *Arch Womens Ment Health* 2015, 18(6):745-759.

13. Hannan J: Older mothers' experiences of postnatal depression. *Br J Midwifery* 2016, 24(1):28-36.

14. Hanley J: The emotional wellbeing of Bangladeshi mothers during the postnatal period. *Community Pract* 2007, 80(5):34-37.

15. Hanley J, Long B: A study of Welsh mothers' experiences of postnatal depression. *Midwifery* 2006, 22(2):147-157.

16. CASP Checklist (Qualitative Research). [accessed 24/05/18]. Available at: [http://docs.wixstatic.com/ugd/dded87_25658615020e427da194a325e7773d42.pdf]

For peer review only - http://bmjopen.bmj.com/site/about/guidelines.xhtml

17. Sandelowski M, Docherty S, Emden C: Focus on qualitative methods. Qualitative metasynthesis: issues and techniques. *Res Nurs Health* 1997, 20(4):365-371.

18. Ziebland S, McPherson A: Making sense of qualitative data analysis: an introduction with illustrations from DIPEx (personal experiences of health and illness). *Med Educ* 2006, 40(5):405-414.

19. Reid PP, Compton WD, Grossman JH, Fanjiang G: The National Academies Collection: Reports funded by National Institutes of Health. In: *Building a Better Delivery System: A New Engineering/Health Care Partnership.* edn. Washington (DC): National Academies Press (US) National Academy of Sciences.; 2005.

20. Ferlie EB, Shortell SM: Improving the quality of health care in the United Kingdom and the United States: a framework for change. *Milbank Q* 2001, 79(2):281-315.

21. Edge D: Falling through the net - black and minority ethnic women and perinatal mental healthcare: health professionals' views. *Gen Hosp Psychiatry* 2010, 32(1):17-25.

22. Edge D: 'It's leaflet, leaflet, leaflet then, "see you later"': black Caribbean women's perceptions of perinatal mental health care. *Br J Gen Pract* 2011, 61(585):256-262.

23. Jomeen J, Glover L, Jones C, Garg D, Marshall C: Assessing women's perinatal psychological health: exploring the experiences of health visitors. *J Reprod Infant Psychol* 2013, 31(5):479-489.

24. Phillips L: Assessing the knowledge of perinatal mental illness among student midwives. *Nurse Educ Pract* 2015, 15(6):463-469.

25. Rothera I, Oates M: Managing perinatal mental health disorders effectively: identifying the necessary components of service provision and delivery. *Psychiatrist* 2008, 32(4):131-133.

26. Boddy R, Gordon C, MacCallum F, McGuinness M: Men's experiences of having a partner who requires Mother and Baby Unit admission for first episode postpartum psychosis. *J Adv Nursing* 2017, 73(2):399-409.

27. Boath EH, Henshaw C, Bradley E: Meeting the challenges of teenage mothers with postpartum depression: Overcoming stigma through support. *J Reprod Infant Psychol* 2013, 31(4):352-369.

28. Chew-Graham C, Chamberlain E, Turner K, Folkes L, Caulfield L, Sharp D: GPs' and health visitors' views on the diagnosis and management of postnatal depression: a qualitative study. *Br J Gen Pract* 2008, 58(548):169-176.

29. Edge D: Perinatal depression and Black Caribbean women: lessons for primary care. *J Prim Health Care* 2007, 17:32-35.

30. Edge D, MacKian SC: Ethnicity and mental health encounters in primary care: help-seeking and help-giving for perinatal depression among Black Caribbean women in the UK. *Ethn Health* 2010, 15(1):93-111.

31. Gardner PL, Bunton P, Edge D, Wittkowski A: The experience of postnatal depression in West African mothers living in the United Kingdom: a qualitative study. *Midwifery* 2014, 30(6):756-763.

32. Glover L, Jomeen J, Urquhart T, Martin CR: Puerperal psychosis–a qualitative study of women's experiences. *J Reprod Infant Psychol* 2014, 32(3):254-269.

33. Lam E, Wittkowski A, Fox JR: A qualitative study of the postpartum experience of Chinese women living in England. *J Reprod Infant Psychol* 2012, 30(1):105-119.

For peer review only

34. Rowan C, McCourt C, Bick D: Provision of perinatal mental health services in two English strategic health authorities: views and perspectives of the multi-professional team. *Evid Based Midwifery* 2010:98.

35. Wan MW, Moulton S, Abel KM: The service needs of mothers with schizophrenia: a qualitative study of perinatal psychiatric and antenatal workers. *Gen Hosp Psychiatry* 2008, 30(2):177-184.

36. Wittkowski A, Zumla A, Glendenning S, Fox J: The experience of postnatal depression in South Asian mothers living in Great Britain: a qualitative study. *J Reprod Infant Psychol* 2011, 29(5):480-492.

37. Wyatt C, Murray C, Davies J, Jomeen J: Postpartum psychosis and relationships: their mutual influence from the perspective of women and significant others. *J Reprod Infant Psychol* 2015, 33(4):426-442.

38. Slade P, Morrell CJ, Rigby A, Ricci K, Spittlehouse J, Brugha TS: Postnatal women's experiences of management of depressive symptoms: a qualitative study. *Br J Gen Pract* 2010, 60(580):e440-e448.

39. Baldwin S, Griffiths P: Do specialist community public health nurses assess risk factors for depression, suicide, and self-harm among South Asian mothers living in London? *Public Health Nurs* 2009, 26(3):277-289.

40. Chew-Graham CA, Sharp D, Chamberlain E, Folkes L, Turner KM: Disclosure of symptoms of postnatal depression, the perspectives of health professionals and women: a qualitative study. *BMC Fam Pract* 2009, 10:7.

41. Coates R, Ayers S, de Visser R: Women's experiences of postnatal distress: a qualitative study. *BMC Pregnancy Childbirth* 2014, 14:359.

42. Patel S, Wittkowski A, Fox JR, Wieck A: An exploration of illness beliefs in mothers with postnatal depression. *Midwifery* 2013, 29(6):682-689.

43. Coates R, de Visser R, Ayers S: Not identifying with postnatal depression: a qualitative study of women's postnatal symptoms of distress and need for support. *J Psychosom Obstet Gynaecol* 2015, 36(3):114-121.

44. Cooke S, Smith I, Turl E, Arnold E, Msetfi RM: Parent perspectives of clinical psychology access when experiencing distress. *Community Pract* 2012, 85(4):34-37.

45. Husain N, Masood Y, Lovell K, Lunat F, Atif N, Waheed W, Rahman A, Mossabir R, Chaudhry N: Group psychological intervention for postnatal depression: a nested qualitative study with British South Asian women. *BMC Womens Health* 2015, 15(1):109.

46. Edge D: 'We don't see black women here': an exploration of the absence of black Caribbean women from clinical and epidemiological data on perinatal depression in the UK. *Midwifery* 2008, 24(4):379–389.

47. McGrath L, Peters S, Wieck A, Wittkowski A: The process of recovery in women who experienced psychosis following childbirth. *BMC Psychiatry* 2013, 13(1):341.

48. Radcliffe P: Substance-misusing women: Stigma in the maternity setting. *Br J Midwifery* 2011, 19(8).

49. Plunkett C, Peters S, Wieck A, Wittkowski A: A qualitative investigation in the role of the baby in recovery from postpartum psychosis. *Clin Psychol Psychother* 2017.

50. Almond P, Lathlean J: Inequity in provision of and access to health visiting postnatal depression services. *J Adv Nurs* 2011, 67(11):2350-2362.

For peer review only

51. Cantwell R, Clutton-Brock T, Cooper G, Dawson A, Drife J, Garrod D, Harper A, Hulbert D, Lucas S, McClure J *et al*: Saving Mothers' Lives: Reviewing maternal deaths to make motherhood safer: 2006-2008. The Eighth Report of the Confidential Enquiries into Maternal Deaths in the United Kingdom. *BJOG* 2011, 118 Suppl 1:1-203.

52. Ross-Davie M, Elliott S, Sarkar A, Green L: A public health role in perinatal mental health: Are midwives ready? *Br J Midwifery* 2006, 14(6):330-334.

53. Stepping Forward to 2020/21: The mental health workforce plan for England July 2017: Mental Health Taskforce strategy [http://www.rcpsych.ac.uk/pdf/FYFV%20Mental%20health%20workforce%20plan%20for%20England%20FINAL.pdf]

54. Implementing the Five Year Forward View for mental health. [accessed 24/05/18]. Available at: [https://www.england.nhs.uk/wp-content/uploads/2016/07/fyfv-mh.pdf]

For peer review only

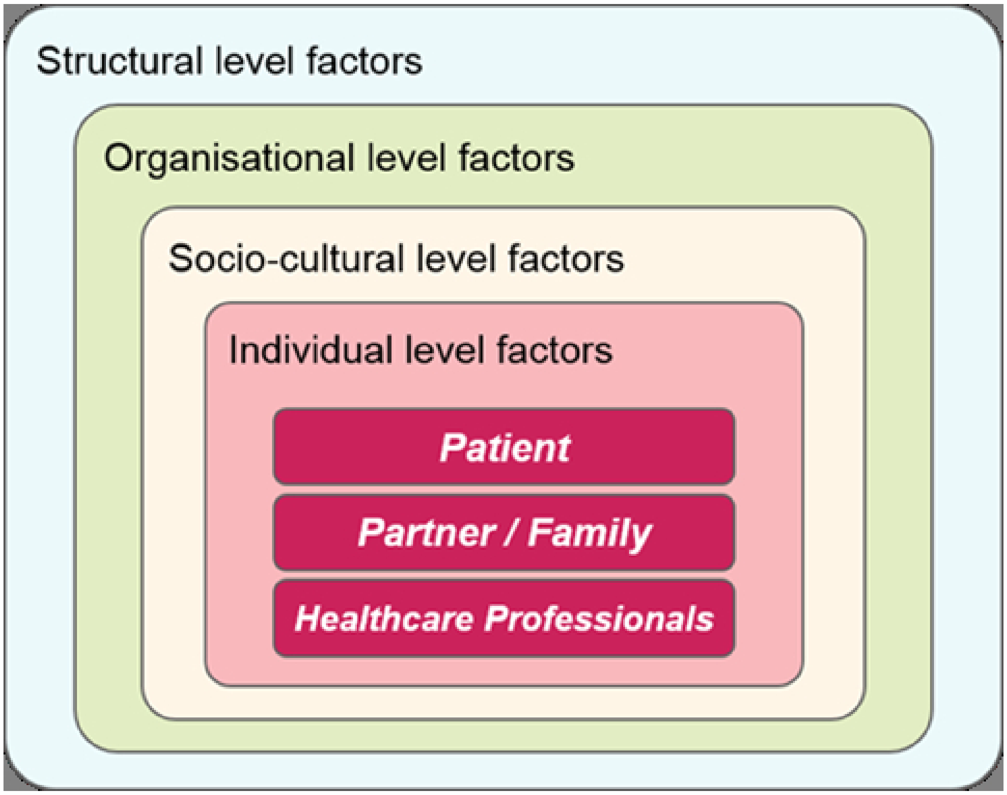

Figure 1: Adapted model showing multi-level conceptual framework for barriers to mental health services in the perinatal period [19,20].

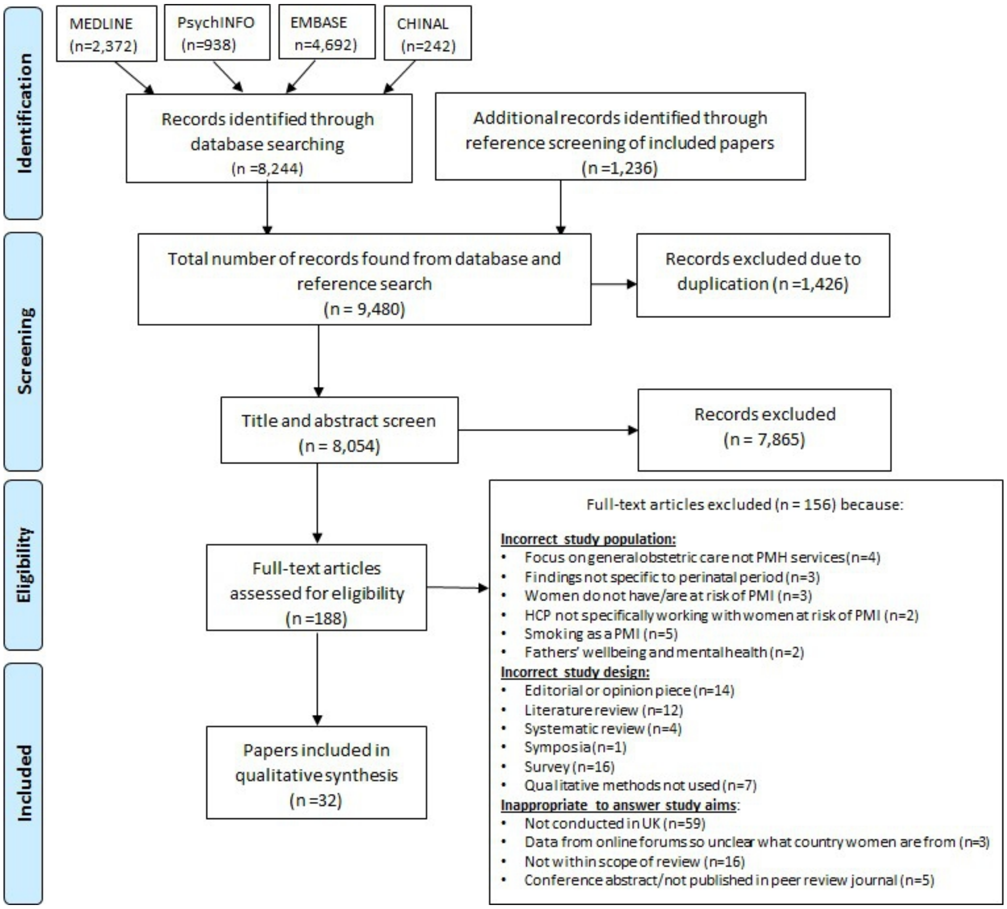

Figure 2: Study Selection

201x180mm (96 x 96 DPI)

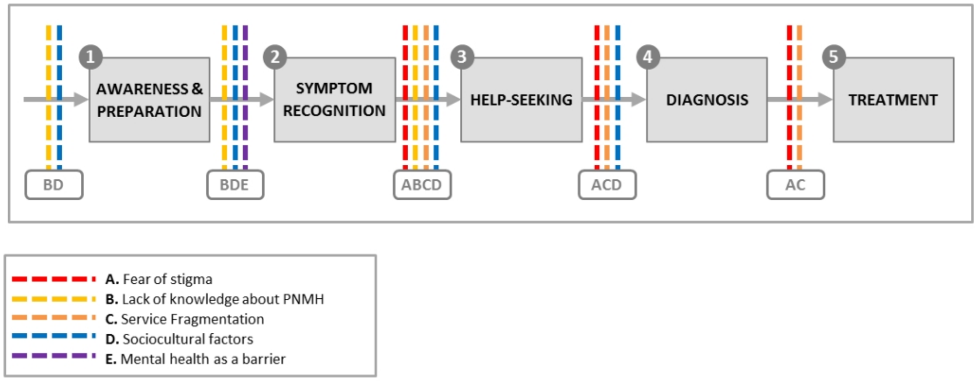

Figure 3: Conceptual model of key barriers in the care pathway to accessing mental health services during the perinatal period

Supplementary Table 1: Comprehensive Summary of Data Extracted from Papers for Review.

	Author /Year	Study Location	Study Aims	Data collection Method	n =	Study Population	Analytic Strategy and interpretative Theory	Key Emergent Themes Relating to Barriers in Care	CASP Score
1	Almond (2011)	Southern England	Understanding equity in prevision of PND services	Observed discussions between HVs and postnatal women and interviews	46	HVs (n=16) managers (n=6) English women (n=12) Bangladeshi women (n=9), cohesion worker (n=2) and mental health practitioner (n=1).	Thematic content data analysis	-Unacceptability of PND screening<br>-Policy not implemented in practice<br>-Cultural and language barriers	Adequate
2	Baldwin (2009)	Inner London	Assessment of South Asian mothers well-being by Specialist community public health nurses	Interviews	8	SCPHN working with South Asian mother	Content analysis	-Poor support networks<br>-Culturally specific barriers<br>-Domestic and emotional abuse	Weak
3	Boath (2013)	Stoke-on-Trent	Teenage mothers' experiences of PND and their care	In-depth interviews	15	First-time mothers aged 16-19 who scored >12 on EPDS	Thematic Framework Analysis	-Consequences of disclosure<br>-Poor continuity of care with preferred HCP<br>-Utilisation of family/friends as alternatives for PNMH services<br>-Poor education about perinatal mental health (PNMH) and mental health services (MHS)<br>-Logistically issues	Strong
4	Boddy (2017)	England	Men's experiences of partner's admission to MBU for first episode PP	In-depth interviews	7	Partners of women receiving inpatient treatment for first episode PP	Interpretive Phenomenological Analysis	-Normalising symptoms<br>- Poor PNMH education<br>-Failures in system to manage emergencies<br>-PNMH as a barrier	Strong
5	Brown (2009)	Coventry, Warwickshire	Improve understanding of PNMH service access	Focus-group discussions	68	Midwives (n=50) and midwifery students (n=18) working with PMIs	Thematic Framework Analysis	-Unacceptability of PND screening<br>-HCP reluctant to diagnose<br>-Midwives have poor training on PNMH<br>-Normalising symptoms<br>-Policy not implemented in practice	Weak

								-Unclear referral pathways<br>-Delays being seem by specialist<br>-Failures in system to manage emergencies	
6	Chew-Graham (2008)	Bristol,<br>Manchester London	Determine GPs' and HVs' roles in management of PND	In-depth interviews	23	GPs (n=(n=19) and HVs (n=14) from RESPOND trial	Inductive thematic analysis approach	-HCPs' reluctant to diagnose<br>-Unacceptability of PND screening<br>-Normalising symptoms<br>-Poor education about PNMH and available treatments<br>-Poor continuity of care and communication<br>-Inappropriate HCP attitudes	Adequate
7	Chew-Graham (2009)*	Bristol, Manchester London	GPs, HVs and<br>women's views on disclosure of PND symptoms	In-depth interviews	28	Women from RESPOND trial scoring >11 on EPDS	Inductive thematic analysis approach	-PNMH diagnosis issues<br>-PNMH as a barrier<br>-Stigma and fear of medications<br>-fear of "bad mum" label<br>-poor opinion of HCPs<br>-Unclear roll of HV<br>-"easier not to ask"<br>-inadequate resources	Adequate
8	Coates (2015)	South East England	PNMH symptoms in new mothers and experiences of assessment	interviews<br>at home (n=15) or via telephone (n=2)	17	Women experiencing distress with baby <1year	Inductive thematic analysis approach<br>-----------------------<br>*Critical realist approach*	-PNMH diagnosis issues<br>-Unacceptability of PND screening<br>-"it is actually alright to ask for help"<br>-Stigma of PNMH<br>-Inadequate resources<br>-Logistically issues	Strong
9	Coates	South East	Experiences of postpartum emotional	interviews at home (n=15) or	17	Women experiencing distress	Interpretive Phenomenological	-Fear of "bad mum" label	Strong

For peer review only

	(2014)	England	distress	via telephone (n=2)		with baby <1year	Analysis	-Normalising symptoms<br>-Waiting for help to come<br>-Poor opinion of HCP<br>-Poor support networks	
10	Cooke (2012)	UK**	Parents' perceptions of barriers to PNMH services	In-depth interviews	7	Parents (>18years) with psychological distress	Thematic Framework Analysis	-Fear of disclosure<br>-Inappropriate HCP attitudes<br>-PNMH as barrier	Weak
11	Edge (2007)	Northern England	Explain low PND service access among Black Caribbean women	In-depth interviews	12	Black Caribbean women, >18years, scoring >12 on EPDS	Grounded theory approach using constant comparison method	-Poor PNMH awareness<br>-Culturally specific barriers<br>-Fear of "bad mum" label<br>-Fear of medication side effects<br>-Fear of disclosure and stigma<br>-Inappropriate HCPs attitudes	Adequate
12	Edge (2008)	North West England	Explain low PND service access among Black Caribbean women their absence from UK perinatal research	In-depth interviews	12	Black Caribbean women, >18years, scoring >12 on EPDS	Thematic Framework Analysis	-Culturally specific barriers<br>-Inappropriate HCP attitudes<br>-Stigma	Strong
13	Edge and MacKian (2010)	North West England	Black Caribbean women's accounts of help-seeking for PND	In-depth interviews	12	Black Caribbean women, >18years, scoring >12 on EPDS	Thematic Framework Analysis<br>-------------------------<br>Draws on social model of depression and MacKian concepts of 'reflexive Communities'	-Culturally specific barriers<br>-Poor PNMH awareness	Strong
14	Edge (2010)	Northern England	HCPs views about PNMH care for BME women	In-depth interviews and Focus Group discussions	42	GPs (n=5), midwives (n=22), midwifery managers (n=5), hospital doctors (n=2), HVs (n=5), volunteers (n=3)	Thematic Framework Analysis	-Physical health over PNMH<br>-Policy not implemented<br>-Diagnostic difficulties	Strong

For peer review only

								-Poor interdisciplinary communication<br>-Unclear referral pathways<br>-Lack of resources<br>-Language barriers	
15	Edge (2011)	Northern England	Black Caribbean women's opinion of PND service access	In-depth interviews	42	Black Caribbean women >18years, with PND or knew someone who had PND	Thematic Framework Analysis	-Previous negative NHS experience<br>-Inadequate resources<br>-Poor support networks<br>-Culture barriers, poor awareness of PNMH	Strong
16	Gardner (2014)	Manchester	UK-based West African mothers experiences of PND	In-depth interviews	6	Black West African women scoring >10 on EPDS and using self-help parenting groups	Interpretive Phenomenological Analysis	-Poor PND awareness<br>-Poor support networks<br>-Lack of religious faith<br>-Logistical issues<br>-Stigma	Adequate
17	Glover (2014)	Northern England	Women's experiences of PP and their causal explanations	In-depth interviews	7	Women accessing specialist psychiatric services due episode of PP <10years ago	Inductive thematic analysis<br>-------------------------<br>Draws on the stress-vulnerability model	-Normalising symptoms<br>-Poor advice from friends<br>-Poor education about PP<br>-Baby as a barrier<br>-Focus on physical health<br>-Stigma	Strong
18	Husain (2015)	Manchester Lancashire	Pilot trial of culturally-adapted PHP for British South Asian women with PND	In-depth interviews (Conducted in Urdu, transcribed in English)	17	British South Asian Women with PND from Bangladesh (n=1) Pakistan (n=14) India (n=2). All participants from ROSHNI-2 trial	Thematic Framework Analysis	-Domestic and emotional abuse<br>-Logistical issues<br>-Language and cultural barriers<br>-Lack of anonymity in therapy<br>-Anxiety about treatment	Adequate

For peer review only

19	Jomeen (2013)	Northern England	HVs' experiences of psychological distress and PMI assessment	Focus Group Discussions	5	HVs attending an optional PNMH training day	Thematic Framework Analysis	-Lack of formal PNMH training<br>-Diagnostic issues<br>-Stigma<br>-Issues with interpreters<br>-Poor opinion of HCPs<br>-Issues with referral pathway<br>-Interdisciplinary communication	Adequate
20	Lam (2012)	Manchester	Investigate Chinese women's postnatal experiences	In-depth interviews (conducted in Chinese and English)	8	UK-based, postnatal, Chinese women. EPDS from 2-17***	Grounded theory approach	-Poor information available<br>-Utilisation of family, friends and online forums as an alternative for PNMH services<br>-Language barrier<br>-Fear of family conflic<br>-Conflicting advice from professionals and family<br>-preoccupied with baby	Adequate
21	McGrath (2013)	North West England	Understanding PP recovery	In-depth interviews conducted via phone (n=1) or face-to-face (n=11)	12	Women with any postnatal psychosis according to International classification of Disease- version 10 (ICD-10)	Grounded theory approach<br>-----------------------<br>Draws on 'Recovery framework' and 'Biopsychosocial models of illness'	-PNMH as a barrier<br>-Fear of "bad mum" label<br>-Stigma	Strong
22	Nicholls (2007)	Sussex	Experiences of postnatal-PTSD in couples relationship	In-depth interviews	12	Couples where either women (n=3), partner (n=1) or both (n=2) fulfilled DSM-IV criteria for postnatal-PTSD ****	Thematic Framework Analysis	-Difficulty of discussing PNMH<br>-Support networks<br>-Previous negative NHS experience	Adequate

For peer review only

23	Patel<br><br>(2013)	North West of England	PND illness beliefs	In-depth interviews	11	Women with PND or depression in pregnancy which continued after birth and scored >10 on EPDS	Grounded Theory<br><br>-------------------------<br><br>Draws on 'Leventhal's self-regulation model of illness beliefs'	-Fear of "bad mum" label<br><br>-Normalising symptoms and unknown cause of symptoms<br><br>-Stigma<br><br>-PNMH as barrier	Strong
24	Phillips (2015)	UK	Student midwives awareness of PMIs	Focus group discussions<br><br>(2 groups lasting 1 hour)	9	Student midwives within 6 months of completing degree.	Thematic Framework Analysis	-Poor resources<br><br>-"easier not to ask"<br><br>-Inadequate education about PNMH	Adequate
25	Plunkett (2017)	North West England	Role of baby in PP recovery from	In-depth interviews phone (n=7) or face-to-face (n=5)	12	Women who had experienced PP. (checked on a "symptom checklist")	Thematic Framework Analysis<br><br>-----------------------<br><br>*****	-Baby as motivating recovery factor<br><br>-Fear of disclosure	Strong
26	Radcliffe (2011)	Three UK hospital trusts	Provider experiences of services access and stigmatisation for substance-misusing women	In-depth interviews and participant<br><br>observations	46	Community midwives, specialist midwives, postnatal ward staff, sonographers (n=22). Perinatal women accessing substance-misuse clinics (n=24)	Grounded Theory<br><br>-----------------------<br><br>Uses a 'social constructionist approach'	-Poor diagnostic tools<br><br>-Stigma<br><br>-Logistical issues (eg. lack of diary to keep appointments)	Weak
27	Rothera (2008)	Trent, Leicestershire, Northampton, Rutland	To highlight issues with PNMH management	In-depth interviews and Focus Group discussions	39	HVs(n=4), Midwife (n=4), GP's (n=3), mental health practitioner (n=2), community psychiatric nurse (n=8), obstetrician (n=5), psychiatrists (n=9), managers (n=4)	Analytic induction and data reduction	-Failure to manage emergencies<br><br>-Policy not implemented<br><br>-Poor HCP PHM education and awareness<br><br>-Interdisciplinary communication	Adequate
28	Rowan (2010)	Southern England	Exploration of policy recommendation implementation	In-depth interviews	8	Managers of maternity services, community midwives, community psychiatric nurses and psychiatrists	Thematic Framework Analysis	-Policy not implemented<br><br>-Issues with catchment areas<br><br>-Poor awareness about PNMH treatments	Adequate

For peer review only

29	Slade (2010)	Trent	Women's experiences of HVs identifying and managing PND (part of PoNDER trial).	In-depth interviews	30	Women in PoNDER trial with 6-week PNDS score >18	template approach	-Poor support networks<br>-Fear of disclosure and stigma<br>-Unclear role of HV and poor training<br>-Poor continuity of care<br>-Inadequate resources<br>-Negative experiences of HCP	Strong
30	Wan<br>(2008)	Manchester Liverpool	Explored providers' perspectives of support needs of mothers with schizophrenia	In-depth interviews	28	Midwives (n=15), obstetric consultant (n=1), psychiatrist (n=1), registered mental health nurses (n=7), manager (n=1) nursery nurse (n=3). Recruited from MBUs (n=9) or antenatal clinics (n=19).	Thematic Framework Analysis	-Fear of consequences<br>-Lack of midwifery training<br>-PNMH as barrier<br>-Interdisciplinary communication<br>-Lack of education about PNMH<br>-Continuity of care<br>-Support networks<br>-Lack of resources	Strong
31	Wittkowski<br>(2011)	Greater Manchester	South Asian mothers' understanding of PND	In-depth interviews	10	South Asian mothers scoring >12 on EPDS	Grounded Theory approach	-Fear of upsetting others, losing job and stigma<br>-Cultural and language barriers<br>-Racial discrimination<br>-Poor PNMH awareness<br>-Poor social support networks<br>-Interdisciplinary communication	Adequate
32	Wyatt (2015)	England	How women and their significant others make sense of PP experiences	In-depth interviews	7	Women with PP and their carers (partners (n=5), family members (n=1) friends (n=1))	Interpretive Phenomenological Analysis	-Poor social support networks<br>-Fear of "bad mum" label<br>-Poor knowledge of PNMH amongst carers	Adequate

For peer review only

								-Feeling guilty for feeling ill	

Table 4.1 Key:

**Quotes from the Chew-Graham 2008 study population were also included in this paper*

***Paper quotes: "Culturally and socio-economically diverse, urban region of UK"*

****This review will only extract data from participants scoring >10 on EPDS*

*****This review will only extract data from the <u>women</u> within the study diagnosed with birth-related PTSD*

******Paper mentions there is limited theory and pre-existing research on this topic so recognises it may not be possible to map findings onto a formal theory*

PRISMA 2009 Checklist

Section/topic	#	Checklist item	Reported on page #
**TITLE**			
Title	1	Identify the report as a systematic review, meta-analysis, or both.	1
**ABSTRACT**			
Structured summary	2	Provide a structured summary including, as applicable: background; objectives; data sources; study eligibility criteria, participants, and interventions; study appraisal and synthesis methods; results; limitations; conclusions and implications of key findings; systematic review registration number.	2-3
**INTRODUCTION**			
Rationale	3	Describe the rationale for the review in the context of what is already known.	4
Objectives	4	Provide an explicit statement of questions being addressed with reference to participants, interventions, comparisons, outcomes, and study design (PICOS).	4
**METHODS**			
Protocol and registration	5	Indicate if a review protocol exists, if and where it can be accessed (e.g., Web address), and, if available, provide registration information including registration number.	5
Eligibility criteria	6	Specify study characteristics (e.g., PICOS, length of follow-up) and report characteristics (e.g., years considered, language, publication status) used as criteria for eligibility, giving rationale.	5
Information sources	7	Describe all information sources (e.g., databases with dates of coverage, contact with study authors to identify additional studies) in the search and date last searched.	5
Search	8	Present full electronic search strategy for at least one database, including any limits used, such that it could be repeated.	5
Study selection	9	State the process for selecting studies (i.e., screening, eligibility, included in systematic review, and, if applicable, included in the meta-analysis).	5
Data collection process	10	Describe method of data extraction from reports (e.g., piloted forms, independently, in duplicate) and any processes for obtaining and confirming data from investigators.	6
Data items	11	List and define all variables for which data were sought (e.g., PICOS, funding sources) and any assumptions and simplifications made.	6-7
Risk of bias in individual studies	12	Describe methods used for assessing risk of bias of individual studies (including specification of whether this was done at the study or outcome level), and how this information is to be used in any data synthesis.	5-6
Summary measures	13	State the principal summary measures (e.g., risk ratio, difference in means).	6
Synthesis of results	14	Describe the methods of handling data and combining results of studies, if done, including measures of consistency (e.g., I^2) for each meta-analysis.	6

For peer review only

For peer review only - http://bmjopen.bmj.com/site/about/guidelines.xhtml

PRISMA 2009 Checklist

Section/topic	#	Checklist item	Reported on page #
Risk of bias across studies	15	Specify any assessment of risk of bias that may affect the cumulative evidence (e.g., publication bias, selective reporting within studies).	5-6
Additional analyses	16	Describe methods of additional analyses (e.g., sensitivity or subgroup analyses, meta-regression), if done, indicating which were pre-specified.	N/A
**RESULTS**			
Study selection	17	Give numbers of studies screened, assessed for eligibility, and included in the review, with reasons for exclusions at each stage, ideally with a flow diagram.	8
Study characteristics	18	For each study, present characteristics for which data were extracted (e.g., study size, PICOS, follow-up period) and provide the citations.	Table 1 in additional documents
Risk of bias within studies	19	Present data on risk of bias of each study and, if available, any outcome level assessment (see item 12).	7
Results of individual studies	20	For all outcomes considered (benefits or harms), present, for each study: (a) simple summary data for each intervention group (b) effect estimates and confidence intervals, ideally with a forest plot.	7
Synthesis of results	21	Present results of each meta-analysis done, including confidence intervals and measures of consistency.	9-13
Risk of bias across studies	22	Present results of any assessment of risk of bias across studies (see Item 15).	7
Additional analysis	23	Give results of additional analyses, if done (e.g., sensitivity or subgroup analyses, meta-regression [see Item 16]).	N/A
**DISCUSSION**			
Summary of evidence	24	Summarize the main findings including the strength of evidence for each main outcome; consider their relevance to key groups (e.g., healthcare providers, users, and policy makers).	14
Limitations	25	Discuss limitations at study and outcome level (e.g., risk of bias), and at review-level (e.g., incomplete retrieval of identified research, reporting bias).	16
Conclusions	26	Provide a general interpretation of the results in the context of other evidence, and implications for future research.	14-16
**FUNDING**			
Funding	27	Describe sources of funding for the systematic review and other support (e.g., supply of data); role of funders for the systematic review.	17

From: Moher D, Liberati A, Tetzlaff J, Altman DG, The PRISMA Group (2009). Preferred Reporting Items for Systematic Reviews and Meta-Analyses: The PRISMA Statement. PLoS Med 6(7): e1000097. doi:10.1371/journal.pmed1000097

For more information, visit: **www.prisma-statement.org**.

For peer review only - http://bmjopen.bmj.com/site/about/guidelines.xhtml

PRISMA 2009 Checklist

For peer review only

BMJ Open

Barriers to Accessing Mental Health Services for Women with Perinatal Mental Illness: Systematic Review and Meta-Synthesis of Qualitative Studies in the UK

Journal:	*BMJ Open*
Manuscript ID	bmjopen-2018-024803.R1
Article Type:	Research
Date Submitted by the Author:	17-Sep-2018
Complete List of Authors:	Megan Sambrook Smith, Megan; King's College London, Institute of Psychiatry, Psychology and Neuroscience, Global Mental Health<br>Lawrence, Vanessa; Kings College, Qualitative Social Sciences<br>Sadler, Euan; Kings College London, Centre for Implementation Science, Health Service & Population Research Department,<br>Easter, Abigail; King's College London, Institute of Psychiatry, Psychology and Neuroscience, Centre for Implementation Science, Health Service & Population Research Department,
**Primary Subject Heading**:	Mental health
Secondary Subject Heading:	Mental health, Health policy
Keywords:	Barriers, Systematic Review, MENTAL HEALTH, Pregnancy, QUALITATIVE RESEARCH

SCHOLARONE™
Manuscripts

Barriers to Accessing Mental Health Services for Women with Perinatal Mental Illness: Systematic Review and Meta-Synthesis of Qualitative Studies in the UK

AUTHORS:

Sambrook Smith, M., MSc[1], Lawrence, V., PhD[2] Sadler, E., PhD[3], Easter, A. PhD[3]

1. Global Mental Health, Institute of Psychiatry, Psychology & Neuroscience, King's College London, 16 De Crespigny Park, London SE5 8AF
2. Health Service and Population Research Department, Institute of Psychiatry, Psychology & Neuroscience, King's College London, PO26, The David Goldberg Centre, De Crespigny Park, London, SE5 8AF
3. Centre for Implementation Science, Health Service & Population Research Department, Institute of Psychiatry, Psychology & Neuroscience, King's College London, De Crespigny Park, London, SE5 8AF

CORRESPONDING AUTHOR

Dr Abigail Easter

Email: abigail.easter@kcl.ac.uk

Phone: 020 7848 5548

WORD COUNT: 4,485

For peer review only

ABSTRACT

Objective: Lack of access to mental health services during the perinatal period is a significant public health concern in the UK. Barriers to accessing services may occur at multiple points in the care pathway. However, no previous reviews have investigated multi-level system barriers or how they might interact to prevent women from accessing services. This review examines women, their family members' and healthcare providers' perspectives of barriers to accessing mental health services for women with perinatal mental illness in the UK.

Design: A systematic review and meta-synthesis of qualitative studies.

Data Sources: Qualitative studies, published between January 2007 and September 2018, were identified in MEDLINE, PsychINFO, EMBASE, and CINAHL electronic databases, hand-searching of reference lists and citation-tracking of included studies. Papers eligible for inclusion were conducted in the UK, used qualitative methods and were focussed on women, family or healthcare providers working with/or at risk of perinatal mental health conditions. Quality assessment was conducted using the Critical Appraisal Skills Programme for qualitative studies.

Results: Of 9,882papers identified, 35 studies met the inclusion criteria. Reporting of emergent themes were informed by an existing multi-level conceptual model. Barriers to accessing mental health services for women with perinatal mental illness were identified at four levels: Individual (e.g. stigma, poor awareness), organisational (e.g. resource inadequacies, service fragmentation), socio-cultural (e.g. language/cultural barriers), and structural (e.g. unclear policy) levels.

Conclusions: Complex, interlinking, multi-level barriers to accessing mental health services for women with perinatal mental illness exist. To improve access to mental healthcare for women with perinatal mental illness multi-level strategies are recommended which address individual, organisational, sociocultural, and structural level barriers at different stages of the care pathway.

Key Words: *barriers; mental health; pregnancy; qualitative studies; systematic review*

For peer review only

STRENGTHS AND LIMITATIONS OF THIS STUDY

- This study provides a comprehensive and robust systematic review of barriers to mental healthcare for women with perinatal mental illness, a key public health issue.
- Robust procedures for systematic reviewing and quality assessment were adopted, in line with PRISMA reporting guidelines.
- Unidentified barriers, specifically those at structural and organisational levels, may remain due to limited and high-quality research specially looking at perceived barriers at these levels
- Due to the wide variability in the context of delivery of perinatal mental health care globally, this review only included studies conducted with the UK. The findings may therefore be less applicable to other healthcare settings.

For peer review only

INTRODUCTION

Approximately 10-20% of women experience mental illness during pregnancy or in the first postpartum year (perinatal period)[1-4]. Perinatal mental illnesses (PMI) are associated with increased morbidity and are a leading cause of maternal death in high-income countries[5, 6]. PMI may also adversely affect psycho-social development of offspring[7], and are associated with significant long-term socio-economic costs[1]. Timely identification and treatment of PMI by trained healthcare professionals (HCPs) is paramount.

The 'Five Year Forward View for Mental Health' aims to transform mental health services (MHS) in the UK, and identifies the need to improve perinatal mental health (PNMH) as a strategic priority for the National Health Service (NHS)[8]. A key recommendation is "by 2020/21, NHS England should support at least 30,000 more women each year to access evidence-based specialist mental healthcare during the perinatal period"[8]. However, in the UK an estimated 60% of women have no access to PNMH services[2] and 38% of women wait over a month to be referred[9]. Inadequate provision of community mental health services, shortages of health visitors (HVs), and midwives and lengthy waiting lists for psychosocial therapies further limit access to MHS for women with PMI[8].

Barriers to care extend beyond inadequate resources[10]. One survey reported that 30% of women withheld negative feelings from HCPs often due to fear of their baby being taken away[2]. Previous reviews highlight that lack of mother-centred antenatal care, stigmatising attitudes towards mental health, and insufficient knowledge among HCPs about PMI contributed to help-seeking delays[11, 12]. There is growing evidence suggesting reasons for difficulties accessing MHS are more complex[13], potentially occur at multiple time points along the care pathway[10] and are compounded by socio-cultural and economic issues[14, 15]. However, no previous review has synthesised evidence on different stakeholder views of where these perceived barriers exist or how they interact to hinder access to MHS during the perinatal period. Identifying where barriers exist is imperative to developing a comprehensive understanding of how to improve access to PNMH care.

This systematic review and meta-synthesis of qualitative studies in the UK examines perceived barriers to accessing MHS for women with PMI from the perspective of women themselves, their family members and HCPs, and provides evidence to support the implementation of the Five Year Forward View Plan.

For peer review only

MATERIALS AND METHODS

We conducted a systematic review and meta-synthesis of qualitative studies.

Search strategy and selection criteria

This systematic review adhered to the PRISMA checklist for reporting findings of systematic reviews and followed a predetermined published protocol registered on the International Prospective Register of Systematic Reviews PROSPERO (ID number CRD42017060389); available at: https://www.crd.york.ac.uk/PROSPERO/display_record.php?RecordID=60389)

The first author (MSS) initially searched *Ovid MEDLINE(R)*, *PsychINFO*, *EMBASE,* and *CINAHL* electronic databases between January 2007 to September 2018 using the following combination of keywords and MeSH terms: ("Perinatal" OR "Pregnancy" OR "Birth") *AND* ("Mental Health" OR "Mental Disorder") *AND* ("Health Service Accessibility OR "Delivery of Health Care") AND ("Qualitative Research" OR "Attitudes of Health Personnel" OR "Health Knowledge, Attitudes, Practice"). See Supplementary File 1 for the full MEDLINE search strategy used. MSS then hand-searched reference lists of included studies and used citation-tracking of these studies in Google Scholar to identify further relevant papers.

We included qualitative studies examining women's, families' and HCPs' perspectives of barriers to accessing MHS for women with mental illness during the perinatal period, published in peer reviewed English language journals. We defined the perinatal period as any time from conception to the first year postnatal. We excluded studies with purely quantitative data or those not conducted in the UK to ensure findings related directly to "Five Year Forward" implementation. For this review, all mental health conditions which occurred during the perinatal period, including poor general mental wellbeing and mental "distress", were included for review. Nicotine addiction and studies exploring barriers to smoking cessation services were excluded from the review.

All papers returned by searches were imported into Endnote (version X7·7·1) and duplicates removed. The first author conducted initial screening and study selection, then two independent reviewers assessed a random 10% sample (n=19) of full text search papers for eligibility (agreement measured using Cohen's Kappa). Abstracts and titles of each paper were then read and full texts retrieved for studies deemed potentially relevant. Two authors (MSS, AE) discussed studies where inclusion was not clear.

Quality appraisal

For peer review only

The quality of all included studies was assessed using the Critical Appraisal Skills Programme (CASP) checklist for qualitative studies, which provides a framework for assessing the quality and rigour of selected studies[16]. The CASP provides 10 questions with a series of prompts to guide the assessment of qualitative papers. A scoring system of 1 mark per question was allocated to provide a useful indicator of quality and enabled comparison across reviewers. Each paper was assessed against the CASP tool and a point for each question was allocated if the criteria had been met. The total CASP scores for all papers were then used to categorise studies as either 'strong' (score >9/10; no methodological issues), 'adequate' (score 9-6/10; no major methodological issues), or 'weak' (score <5/10; major methodological issues) quality. Quality assessment was carried out by the first author and then 20% (n=6) by two additional reviewers (ES, AE). Agreement was calculated using Cohen's Kappa and disagreements were resolved by discussion between MSS, ES and AE. The use of quality assessments scores is contentious in qualitative research due to difficulties in applying one criterion to multiple qualitative methodologies and journals requesting different reporting requirements. Therefore, we adopted an inclusive approach and studies with a low CASP score were not excluded from the review.

Data Extraction and synthesis approach

A meta-synthesis approach was used to synthesise findings from qualitative studies[17]. The approach was chosen to improve understanding and conceptual development greater than that attained from the individual studies alone[17]. We constructed data extraction tables to record key characteristics and summary findings from included studies (Supplementary Table 1). All raw data extracted from each paper was purely in the form of direct quotes and patient sociodemographic data and did not include authors interpretation of their findings. A constant comparison method approach was used to identify emerging themes and related subthemes, including discordant themes, looking for similarities and differences in stakeholder perspectives across the data extracted from all papers. These were then graphically displayed as a 'conceptual map' to visually display themes and explore relationships between themes and related subthemes[18]. All authors met regularly to discuss the emerging themes.

A theoretical multi-level conceptual framework based on the "delivery systems" model (figure 1)[19, 20] was subsequently used to help organise, report and interpret meta-synthesis findings. This adapted model, based on Ferlie and Shortell's "Framework for change"[20] in combination with Reid's "delivery system"[19], was created through discussion between two authors (MSS and ES) after reviewing included papers. This was to allow for specific individual, organisational, socio-

For peer review only

cultural and structural level factors (e.g. policy and politico-economic factors) to be drawn out of the analysis and provided a theory-driven approach.

Figure 1 here

PATIENT AND PUBLIC INVOLVEMENT

The development of the research questions for this study was directly informed by the NHS 'Five Year Forward View for Mental Health' [8]. The priorities laid out in the Five Year Forward View were established by an independent Mental Health Taskforce, which brought together health and care leaders, service users and experts in the field. The findings will be disseminated widely to service user groups and voluntary organisations.

For peer review only

RESULTS

In total, we identified 9,882articles, of which 30 qualitative studies met the eligibility criteria. A further five papers were identified through citation tracking. Therefore a total of 35 papers, reporting on 32 studies, were included for review (figure 2).

Supplementary Table 1 provides a summary table of study characteristics of included qualitative studies. Postnatal Depression (PND; n=13) and poor reported mental well-being (n=10) were the most commonly studied PMIs. Other studies commented on rare postnatal outcomes (e.g postpartum psychosis and birth-related PTSD), antenatal anxiety and perinatal substance misuse. Data collection was mostly via semi-structured interviews (n=30) with two studies using non-participant observations and seven studies utilising focus groups[10, 21-26] [10, 21-26]]]. In 21 papers, the study population was women with PMI and 13 papers focused on HCPs working with these women, with four studies including family and friends as research participants.

Overall, 88% of the studies were deemed either high (n=18) or moderate quality (n=13). CASP scores below 5 and graded as "weak" quality (n=4) were associated with poor reflexivity (e.g. researchers not considering how their own personal values affected the data collection) and non-rigorous data analysis methods. Supplementary Table 2 provides a full summary of quality ratings for each of the included studies. A low level of agreement in a sample of included studies between reviewers and the original CASP score given by MSS was seen (reviewer 1 (K)=0·2; reviewer 2 (K)=0). This was partly due to poor reporting of information resulting in difficulties assessing the true methodological quality of the studies. Considering these discrepancies, a discussion between MSS, AE and ES took place to reach a consensus on study quality of included studies.

Figure 2 here

Meta-synthesis of findings: Multi-level barriers to Mental Health Services

Barriers to accessing MHS for women with PMI related to a wide range of complex factors. Drawing on Reid's delivery system model[19], such factors operated on multiple-levels: individual (knowledge, attitudes and individual characteristics of women, their families and HCPs), organisational (organisational characteristics, service access, and inadequacy of resources), socio-cultural (family support, wider social support networks, and cultural attitudes), and structural (unclear policy) levels.

Individual level factors

Lack of knowledge about Perinatal Mental Health

For peer review only

Poor PMI awareness and knowledge among HCPs and women was cited in 14 studies as a barrier to accessing appropriate care[22, 27-39] . Unfamiliarity with the concept of PNMH and the signs and symptoms of mental illness, as well as a perceived lack of open discussion between HCPs and family members were reported as common issues for women[22, 27, 28, 30-34, 37, 38]. One woman said:

> "I didn't really know the meaning of it [postpartum depression] …I could have detected it earlier if someone had explained to me what your first symptoms were, but nobody told me." (Teenage mother with PND)[28]

HCPs similarly reported poor knowledge of PMIs in a number of studies[29, 35, 36] which was often attributed to inadequate training opportunities[10, 23-25, 35, 36, 40]. One student midwife commented that "mental health is very challenging; we are not trained to give mental healthcare"[24]. Student midwives also highlighted gaps in the training curriculum as "there was only one lecture on mental health [and] no formal training"[10, 24, 39].

Family members and friends of women with PMI played an important role in detecting signs and symptoms of illness. However, several studies found that family and friends could also hinder women from disclosing a mental illness to HCPs, often due to perceived stigma, leading to delays in seeking professional support [22, 27, 28, 33, 34, 37, 38, 41-43]. Family members also described feeling unable to recognise deteriorating signs and symptoms and therefore were unable to provide effective support[29]. Normalising symptoms of mental illness due to pregnancy and motherhood was highlighted in several studies as a way of explaining changes in maternal behaviour[10, 27, 29, 33, 43-45]. Women with PMI commonly attributed symptoms (e.g. low mood and self-esteem) to tiredness or hormones, whereas partners and HCPs tended to dismiss such symptoms as part of the normal pregnancy experience. For example, one male spouse of a women with postpartum psychosis commented:

> "I… didn't really see the more acute signs because A. I'm not experienced in them and B. I knew there was something up but I put it down to her being absolutely over exhausted."[27]

Insufficient knowledge among HCPs about care pathways for women with PMI was reported in eleven studies[22, 26-31, 35, 46-48]. In three of these studies, midwives, and HVs voiced a perceived lack of confidence, knowledge and skills to refer women to appropriate services[10, 21, 23] and obstetricians in one of the studies spoke about not knowing what local services were currently available[10], resulting in perceived delays in accessing services especially in complex cases[23] and emergencies[10, 25, 27].

Negative attitudes towards mental illness

For peer review only

Stigma, guilt, and shame associated with being given a PMI diagnosis and treatment was reported by women in 11 papers[23, 30, 32, 33, 40, 42, 44, 46, 49-51]. Sometimes women expressed feeling guilty about being ill at a time when happiness was expected[38, 44, 50]. Commonly concerns centred around negative consequences and stigma of disclosure, such as being labelled a 'bad mum'[30, 38, 42-45, 50], not wanting to upset other family members[34, 38], or not fulfilling perceived social expectations of motherhood[28, 30, 34, 36, 37, 40, 47, 52]. Women from minority groups particularly felt they were at risk of stigmatising attitudes from HCPs and the public due to cultural differences in social expectations[22, 28, 30, 37, 49]. For example, one Pakistani woman with PND said:

"There is a huge stigma of being mentally ill in the public, but for us Asians there is a double disadvantage. I really fear that work will find out." [37]Similarly, HCPs were sometimes reluctant to formally recognise symptoms related to PND because they did not want to impose labels on women[10, 29]. This was emphasised in one study among midwives who reported feeling uncomfortable about recording such concerns in women's medical notes which family members potentially had access to[24]. Furthermore, HCPs in six studies reported that women had refused treatment because of concerns around taking psychotropic medications, including the perceived stigma and feelings of failure as a good mother, fear of harm to their babies, and fears of dependence on medications and associated side effects[22, 23, 29, 30, 42, 48].

Organisational level factors

Inadequate resources

Inadequate resources in terms of staff shortages and limited service provision were reported by HCPs as key organisational barriers to providing effective services for women with PMI in a number of studies[21, 22, 24, 36, 39, 40, 42, 46]. Midwives spoke about not having sufficient time to build rapport with women with PMI and some were "criticised as slow" by other HCPs if they were perceived to take more time[24]. In one study, a student midwife felt even if "information and knowledge can be there, there is no time" to provide support for women to access PNMH services, which shaped a sense of frustration[24]. Other logistical barriers related to organisational factors included limited childcare facilities and integration of babies within the therapy session resulting in non-attendance at appointments[45, 48]. There seemed to be mixed responses from health professions as to why the babies could not be integrated within the therapy sessions:

> "If you're doing some sort of therapy, perhaps trauma work, I don't think it would be appropriate to have a baby in the session because the mum's going to get so upset"(CBT therapist)[45]

For peer review only

Fragmented services: role clarity and conflict

Perceptions of poor continuity of care and not knowing which HVs to contact were reasons for non-disclosure among women with PMI in five studies[10, 28, 29, 36, 40]. Some HCPs described how a perceived lack of specialist services and long waiting lists adversely affected access to appropriate care[10, 25]. In one further study, variations in service organisation across different NHS Trusts in the UK were viewed by a range of HCPs to cause particular challenges in the healthcare system, and were perceived to compromise the creation of a "completely secure safety net" of care[35]. For example, one GP commented:

> "We have terrible trouble with HVs… because the HVs are now sectorised, we have to liaise with about 12 different HVs. It is just a nightmare! Deeply unsatisfactory! It's not the HVs' fault — it's the system."[21]

Perceptions of fragmented services among HCPs were considered to cause problems with inter-disciplinary communication between professional groups, which hindered access of care for women with PMI[21, 23, 25, 26, 29, 36, 37]. Communication was seen as particularly poor between primary care staff and mental health services[25, 29, 37, 42], in emergency situations[10, 27, 42] and during the handover of care from midwives to HVs[23, 36]. This left HVs in one study feeling frustrated and unsupported by other colleagues[23]. HVs in two further studies emphasised how fragmented services created confusion about the HV's role within the referral pathway[40, 42]. Similarly, women were also confused about the HV role in supporting them to access appropriate care[40, 42], particularly in terms of liaising with social care providers. Women in a further study voiced uncertainty regarding knowing who was the most appropriate HCP to approach to access PND services[37]. For example, one woman with PND said:

> "My GP says go the HV and HV says go to GP. I don't know what to do, I need help, don't know where to go, or who to turn to"[37]

Socio-cultural level factors

Language barriers

Language as a barrier to accessing MHS and care was similarly reported by both mothers and HCPs in a third of included studies[21, 22, 30, 31, 34, 37, 41, 48, 49, 53]. Women from minority ethnic backgrounds felt they encountered significant barriers when requesting translators[34, 53]. For example, one Chinese woman said:

For peer review only

> "When the midwife visits, I can only speak the sentences about requesting a translator … They said that this kind of service is limited … that is what is difficult being Chinese – language barrier."[34]

In one study midwives and HVs seemed to underestimate the importance of translators for such women or were frustrated at the extra work required to arrange such services[53]. This sometimes resulted in the overreliance on partners of women with PMI for translation which resulted in inaccuracies and ambiguity with exactly what the women wished to communication[53]. For example, one HV commented:

> "Because sometimes they say loads and then they come back saying, 'She said no'. I know that they've probably done it in shorthand." (HV)[53]

Differences in Cultural Values

The relationship between cultural attitudes, access to MHS and associated challenges this raised for women with PMI was an emerging theme in several studies[22, 30, 31, 34, 37, 41, 48, 49, 53]. The main barriers to accessing appropriate care for women from Black minority ethnic (BME) groups included dismissing mental health as a "something the doctors made up"[30], being unable to disclose feelings due to differences in ethnic backgrounds of HCPs[22, 30, 31, 49], and not receiving perceived culturally appropriate support (e.g. no available female doctors)[37]. With regards to the need to access specialist services, some women from minority ethnic groups in three studies spoke about the importance of the cultural competency of HCPs to promote and encourage help-seeking [30, 36, 53]. For example, one woman felt that she was met with culturally insensitive attitudes from her consultant:

> "I went to see the consultant about my hypertension a couple of weeks ago…and when I told him [about HV's 'diagnosis'], he said, 'you haven't got postnatal depression. You're too cheerful and bright and laughing" (BME woman with PND)[49]

Structural Level Factors

Unclear Policy around appropriate and acceptable use of assessment tools

A key theme among HCPs was the need for clearer policies to be implemented to address potential barriers to accessing MHS's for women with PMI. Polices discussed in various papers centred on the recommended use of appropriate assessment tools for diagnosis of PMI[10, 21, 23, 25, 26, 29, 35, 46, 51, 53] and pathways of care[10, 21, 23, 45]. HCPs frequently expressed negativity towards the use of existing assessment tools (such as the Edinburgh Postnatal Depression Scale (EPDS))[10, 21,

For peer review only

23, 25, 29, 35, 46, 51, 53]. Midwives, HVs and GPs agreed that such screening tools were currently unsatisfactory[21, 23], and inconsistent usage was perceived to result in many women being missed in the system[46].

In contrast, women with PMI in four studies found the process of assessment therapeutic as they felt their symptoms were being taken seriously and had received formal recognition from professionals that they were unwell[40, 45, 47, 53]. However, poor implementation of assessment tools by HCPs shaped negative perceptions among women in several studies of the care received. These included feeling that assessments were tick box exercises[45, 46], conducted at inappropriate times[40, 46], and that findings sometimes did not reflect their experiences of PMI[45, 46]. Lack of resources, treatment options, and poor knowledge of referral pathways also led to perceived ethical concerns amongst one HV who said:

> "In an ideal world we'd want to pick them up and then offer them more support, but we can't do that. So there's almost this ethical dilemma of well is there any point in identifying them if you can't do anything with them other than send them to the GP for antidepressants, which isn't good, you know?" (HV)[29]

DISCUSSION

This review has identified multi-level barriers to accessing MHS for women with PMI in the UK during the perinatal period. In summary, we found that negative attitudes towards diagnosis and treatment of PMIs resulted in women avoiding help-seeking and reinforced feelings of stigma and guilt. Lack of PNMH knowledge among HCPs, women and their families led to poor recognition of symptoms, delayed referrals, and confusion over the role of the HV. Organisational level factors such as inadequate resources, fragmentation of services, and poor interdisciplinary communication compounded these individual level issues. Structural factors (especially poor policy implementation) and socio-cultural factors (e.g. language barriers) also caused significant barriers to accessing services for this group of women.

Based on the findings from this review, we propose a conceptual model to explain where these barriers fit within the care pathway for perinatal woman requiring MHS (figure 3).

Figure 3 here

The first stage of the care pathway involves identification of high-risk women and provision of general PNMH information to all pregnant women. We found that a key barrier to implementing this is poor general knowledge and education about MHS among women with PMI, their families, and

For peer review only

HCPs[22, 27-39], especially among teenage[28], BME[21, 22, 30-32, 49] and South-Asian mothers[37, 41, 48]. Evidence has shown that midwives, who are best placed to discuss perinatal mental health risk with pregnant women, receive inadequate PNMH education and training[54][55] with a high proportion reporting receiving no mental health training at all[54]. "Stepping Forward to 2020"[56], which outlines methods to achieve the "Five Year Forward View For Mental Health"[8], recommends development and implementation of a competency framework for staff. Our review supports this proposal, however there is also evidence from our review that women and their families had poor PNMH knowledge, highlighting the need for broader approaches to improve knowledge in these groups. Alongside this, innovative national public health campaigns, such as the "Everyone's Business Campaign led by the Maternal Mental Health Alliance, are important for raising awareness and reducing stigmatising attitudes felt by women, which act as barriers at stages 3-5 of the care pathway.

To provide appropriate support HCPs need to correctly identify common PNMH symptoms in distressed women and acknowledge symptoms in mothers who are not actively seeking help via routine mental health assessments (stage 2). Barriers to implementing this identified in this review include difficulties women have in differentiating between PNMH symptoms and "normal" pregnancy experiences, with some women lacking the insight and capacity to recognise they were unwell, especially those with PP[27, 33] and severe PND[29, 44]). Family members also play an important role in recognising symptoms of PMI[22, 27, 28, 33, 34, 37, 38, 41-43], however this was especially difficult for women from minority ethnic backgrounds, for example, those from Chinese[34], BME[30, 31, 49] and for South Asian women[37, 41, 48], with such subgroups largely not familiar with PNMH and presenting symptoms. High quality and culturally sensitive information about PNMH needs to be provided to each woman to highlight differences between perceived normal pregnancy changes and PNMH symptoms. Information needs to also include red flag signs, information for concerned family members, HCP contact information, and emergency protocols. Such resources should be available in multiple languages and adapted for cultural relevance.

Most studies in this review focused on factors influencing help-seeking among women with PMI (stage 3). Women often received conflicting advice about who best to approach in the system due to poor interdisciplinary communication between HCPs about their specific roles in the management of women with PMI[21, 23, 25, 29, 36, 37]. The "Stepping Forward" report proposes to create new job roles within PNMH care (e.g. psychological well-being practitioner) to address this issue[56]. However, to improve access to services there is also a need for clearer role clarification and understanding of referral pathways.

For peer review only

This review found that barriers preventing diagnosis (stage 4) of PMI among women mostly related to two factors: (1) issues with screening and diagnostic tools; and (2) HCPs reluctance to label mental health conditions due to fear of stigmatisation of the woman with PMI. Both factors, coupled with wider structural and organisational level barriers (e.g. limited resources) shaped complex ethical issues related to the diagnosis and routine screening of PMI for this group of women. The final stage of the care pathway relates to receiving appropriate treatment for women with PMI. Increasing the number of community PNMH services and Mother and Baby Units will provide much needed additional resources[57], however it is clear from this review that for women to access these services implementation strategies that address barriers at earlier stages of the care pathway are crucial.

We acknowledge several possible limitations of our review. One possible limitation is that unidentified barriers, specifically those at structural and organisational levels, may remain due to limited research in these areas. Secondly, although most studies within the review were deemed of 'strong' or 'adequate' quality, there were gaps in reporting especially in terms of under-reporting of possible researcher bias during data collection and analysis. Another potential limitation was that only one reviewer independently reviewed the quality of all included studies (with 10% cross-checked by two reviewers). However, the use of quality appraisal methods in qualitative evidence is contentious and we did not exclude articles on this basis. Furthermore, including papers in the review deemed poor quality did not affect the analysis as extracted themes did not seem to differ according to CASP scores. Small sample sizes in some of the included studies were another issue in terms of drawing out wider implications. However, the meta-synthesis approach we used enabled the pooling of emerging themes and related sub-themes, thus enhancing the robustness and credibility of the results from this review.

In conclusion, this systematic review and meta-synthesis of qualitative studies found multi-level barriers to accessing MHS for women with PMI in the UK. To make tangible and sustainable improvements to expand access to care for this group of patients, we advocate changes need to be implemented at several stages within the proposed care pathway, with specific attention given to targeting key barriers to accessing MHS for women with PMI. Furthermore, in increasing the number of specialist PNMH services and staff it is also vital that strategies are used to reduce individual, organisational, socio-cultural, and structural level barriers that women with PMI are facing in accessing MHS services in the UK. It will not be until these barriers are addressed that the targets outlined in the "Five Year Forward View for Mental Health" can be optimally met.

Figure Legends

For peer review only

Figure 1: Adapted model showing multi-level conceptual framework for barriers to mental health services in the perinatal period [19,20]

Figure 2: Study Selection

Figure 3: Conceptual model of key barriers in the care pathway to accessing mental health services during the perinatal period

Supplementary File 1: Full Search Strategy for Medline via Ovid

Supplementary Table 1: Comprehensive summary of data extracted from papers for review

Supplementary Table 2: Summary of CASP tool used for quality appraisal of Qualitative Data

For peer review only

AUTHORS CONTRIBUTIONS

AE was responsible for the original conception and design of the work, with significant contributions from MSS, ES and VL. MSS was primarily responsible for conducting the review and data analysis, with quality appraisal checks on a sample of studies conducted by AE and ES. All authors made significant contributions to interpretation of the study findings. MSS produced the initial manuscript draft and further redrafts were critically revised and approved by all authors.

ACKNOWLEDGEMENTS

The research was supported by the National Institute for Health Research (NIHR) Collaboration for Leadership in Applied Health Research and Care (CLAHRC) South London at King's College Hospital NHS Foundation Trust, and the NIHR Biomedical Research Centre, Guy's and St Thomas' NHS Foundation Trust and King's College London, UK. The views expressed are those of the authors and not necessarily those of the NHS, the NIHR or the Department of Health.

FUNDING

AE and ES are funded through individual King's Improvement Science Fellowship awards. King's Improvement Science is part of the NIHR CLAHRC South London and comprises a specialist team of improvement scientists and senior researchers based at King's College London. Its work is funded by King's Health Partners (Guy's and St Thomas' NHS Foundation Trust, King's College Hospital NHS Foundation Trust, King's College London and South London and Maudsley NHS Foundation Trust), Guy's and St Thomas' Charity, the Maudsley Charity and the Health Foundation.

DECLARATION OF INTEREST

None

DATA SHARING STATEMENT

This study is a systematic review, all data included within the present study has been previously published and in the public domain.

For peer review only

References

1. Leight KL, Fitelson EM, Weston CA, Wisner KL: **Childbirth and mental disorders**. *Int Rev Psychiatry* 2010, **22**(5):453-471.

2. **Costs of perinatal mental health problems Centre for Mental Health: Personal Social Services Research Unit** [http://eprints.lse.ac.uk/59885/1/__lse.ac.uk_storage_LIBRARY_Secondary_libfile_shared_repository_Content_Bauer%2C%20M_Bauer_Costs_perinatal_%20mental_2014_Bauer_Costs_perinatal_mental_2014_author.pdf.]

3. Howard LM, Molyneaux E, Dennis CL, Rochat T, Stein A, Milgrom J: **Non-psychotic mental disorders in the perinatal period**. *Lancet* 2014, **384**(9956):1775-1788.

4. O'Hara MW, Wisner KL: **Perinatal mental illness: definition, description and aetiology**. *Best Pract Res Clin Obstet Gynaecol* 2014, **28**(1):3-12.

5. Wilkinson H: **Saving mothers' lives. Reviewing maternal deaths to make motherhood safer: 2006-2008**. *BJOG* 2011, **118**(11):1402-1403; discussion 1403-1404.

6. **Saving Lives, Improving Mothers' Care Lessons learned to inform future maternity care from the UK and Ireland Confidential Enquiries into Maternal Deaths and Morbidity 2009-2012** [https://www.npeu.ox.ac.uk/downloads/files/mbrrace-uk/reports/Saving%20Lives%20Improving%20Mothers%20Care%20report%202014%20Full.pdf.]

7. Stein A, Pearson RM, Goodman SH, Rapa E, Rahman A, McCallum M, Howard LM, Pariante CM: **Effects of perinatal mental disorders on the fetus and child**. *Lancet* 2014, **384**(9956):1800-1819.

8. **The five year forward for Mental Health: A report from the independent Mental Health Taskforce to the NHS in England February 2016. : Mental Health Taskforce strategy** [https://www.england.nhs.uk/wp-content/uploads/2016/02/Mental-Health-Taskforce-FYFV-final.pdf]

9. **Maternal Mental Health –Women's Voices** [https://www.rcog.org.uk/globalassets/documents/patients/information/maternalmental-healthwomens-voices.pdf.]

10. Brown M, Mills N, McCalmont C, Lees S: **Perinatal mental health services: the problems in identifying and addressing mental health issues in pregnant women**. *Primary Health Care* 2009, **19**(8):36-39.

11. Dennis CL, Chung-Lee L: **Postpartum depression help-seeking barriers and maternal treatment preferences: a qualitative systematic review**. *Birth* 2006, **33**(4):323-331.

12. Megnin-Viggars O, Symington I, Howard LM, Pilling S: **Experience of care for mental health problems in the antenatal or postnatal period for women in the UK: a systematic review and meta-synthesis of qualitative research**. *Arch Womens Ment Health* 2015, **18**(6):745-759.

13. Hannan J: **Older mothers' experiences of postnatal depression**. *Br J Midwifery* 2016, **24**(1):28-36.

14. Hanley J: **The emotional wellbeing of Bangladeshi mothers during the postnatal period**. *Community Pract* 2007, **80**(5):34-37.

15. Hanley J, Long B: **A study of Welsh mothers' experiences of postnatal depression**. *Midwifery* 2006, **22**(2):147-157.

For peer review only

16. **CASP Checklist (Qualitative Research)** [http://docs.wixstatic.com/ugd/dded87_25658615020e427da194a325e7773d42.pdf]

17. Sandelowski M, Docherty S, Emden C: **Focus on qualitative methods. Qualitative metasynthesis: issues and techniques**. *Res Nurs Health* 1997, **20**(4):365-371.

18. Ziebland S, McPherson A: **Making sense of qualitative data analysis: an introduction with illustrations from DIPEx (personal experiences of health and illness)**. *Med Educ* 2006, **40**(5):405-414.

19. Reid PP, Compton WD, Grossman JH, Fanjiang G: **The National Academies Collection: Reports funded by National Institutes of Health**. In: *Building a Better Delivery System: A New Engineering/Health Care Partnership.* edn. Washington (DC): National Academies Press (US)

National Academy of Sciences.; 2005.

20. Ferlie EB, Shortell SM: **Improving the quality of health care in the United Kingdom and the United States: a framework for change**. *Milbank Q* 2001, **79**(2):281-315.

21. Edge D: **Falling through the net - black and minority ethnic women and perinatal mental healthcare: health professionals' views**. *Gen Hosp Psychiatry* 2010, **32**(1):17-25.

22. Edge D: **'It's leaflet, leaflet, leaflet then, "see you later"': black Caribbean women's perceptions of perinatal mental health care**. *Br J Gen Pract* 2011, **61**(585):256-262.

23. Jomeen J, Glover L, Jones C, Garg D, Marshall C: **Assessing women's perinatal psychological health: exploring the experiences of health visitors**. *J Reprod Infant Psychol* 2013, **31**(5):479-489.

24. Phillips L: **Assessing the knowledge of perinatal mental illness among student midwives**. *Nurse Educ Pract* 2015, **15**(6):463-469.

25. Rothera I, Oates M: **Managing perinatal mental health disorders effectively: identifying the necessary components of service provision and delivery**. *Psychiatrist* 2008, **32**(4):131-133.

26. Evans K, Morrell CJ, Spiby H: **Women's views on anxiety in pregnancy and the use of anxiety instruments: a qualitative study**. *Journal of reproductive and infant psychology* 2017, **35**(1):77-90.

27. Boddy R, Gordon C, MacCallum F, McGuinness M: **Men's experiences of having a partner who requires Mother and Baby Unit admission for first episode postpartum psychosis**. *J Adv Nursing* 2017, **73**(2):399-409.

28. Boath EH, Henshaw C, Bradley E: **Meeting the challenges of teenage mothers with postpartum depression: Overcoming stigma through support**. *J Reprod Infant Psychol* 2013, **31**(4):352-369.

29. Chew-Graham C, Chamberlain E, Turner K, Folkes L, Caulfield L, Sharp D: **GPs' and health visitors' views on the diagnosis and management of postnatal depression: a qualitative study**. *Br J Gen Pract* 2008, **58**(548):169-176.

30. Edge D: **Perinatal depression and Black Caribbean women: lessons for primary care**. *J Prim Health Care* 2007, **17**:32-35.

31. Edge D, MacKian SC: **Ethnicity and mental health encounters in primary care: help-seeking and help-giving for perinatal depression among Black Caribbean women in the UK**. *Ethn Health* 2010, **15**(1):93-111.

32. Gardner PL, Bunton P, Edge D, Wittkowski A: **The experience of postnatal depression in West African mothers living in the United Kingdom: a qualitative study**. *Midwifery* 2014, **30**(6):756-763.

For peer review only

33. Glover L, Jomeen J, Urquhart T, Martin CR: **Puerperal psychosis–a qualitative study of women's experiences**. *J Reprod Infant Psychol* 2014, **32**(3):254-269.

34. Lam E, Wittkowski A, Fox JR: **A qualitative study of the postpartum experience of Chinese women living in England**. *J Reprod Infant Psychol* 2012, **30**(1):105-119.

35. Rowan C, McCourt C, Bick D: **Provision of perinatal mental health services in two English strategic health authorities: views and perspectives of the multi-professional team**. *Evid Based Midwifery* 2010:98.

36. Wan MW, Moulton S, Abel KM: **The service needs of mothers with schizophrenia: a qualitative study of perinatal psychiatric and antenatal workers**. *Gen Hosp Psychiatry* 2008, **30**(2):177-184.

37. Wittkowski A, Zumla A, Glendenning S, Fox J: **The experience of postnatal depression in South Asian mothers living in Great Britain: a qualitative study**. *J Reprod Infant Psychol* 2011, **29**(5):480-492.

38. Wyatt C, Murray C, Davies J, Jomeen J: **Postpartum psychosis and relationships: their mutual influence from the perspective of women and significant others**. *J Reprod Infant Psychol* 2015, **33**(4):426-442.

39. McGookin A, Furber C, Smith DM: **Student midwives' awareness, knowledge, and experiences of antenatal anxiety within clinical practice**. *Journal of reproductive and infant psychology* 2017, **35**(4):380-393.

40. Slade P, Morrell CJ, Rigby A, Ricci K, Spittlehouse J, Brugha TS: **Postnatal women's experiences of management of depressive symptoms: a qualitative study**. *Br J Gen Pract* 2010, **60**(580):e440-e448.

41. Baldwin S, Griffiths P: **Do specialist community public health nurses assess risk factors for depression, suicide, and self-harm among South Asian mothers living in London?** *Public Health Nurs* 2009, **26**(3):277-289.

42. Chew-Graham CA, Sharp D, Chamberlain E, Folkes L, Turner KM: **Disclosure of symptoms of postnatal depression, the perspectives of health professionals and women: a qualitative study**. *BMC Fam Pract* 2009, **10**:7.

43. Coates R, Ayers S, de Visser R: **Women's experiences of postnatal distress: a qualitative study**. *BMC Pregnancy Childbirth* 2014, **14**:359.

44. Patel S, Wittkowski A, Fox JR, Wieck A: **An exploration of illness beliefs in mothers with postnatal depression**. *Midwifery* 2013, **29**(6):682-689.

45. Millett L, Taylor BL, Howard LM, Bick D, Stanley N, Johnson S: **Experiences of Improving Access to Psychological Therapy Services for Perinatal Mental Health Difficulties: a Qualitative Study of Women's and Therapists' Views**. *Behavioural and cognitive psychotherapy* 2018, **46**(4):421-436.

46. Coates R, de Visser R, Ayers S: **Not identifying with postnatal depression: a qualitative study of women's postnatal symptoms of distress and need for support**. *J Psychosom Obstet Gynaecol* 2015, **36**(3):114-121.

47. Cooke S, Smith I, Turl E, Arnold E, Msetfi RM: **Parent perspectives of clinical psychology access when experiencing distress**. *Community Pract* 2012, **85**(4):34-37.

48. Husain N, Masood Y, Lovell K, Lunat F, Atif N, Waheed W, Rahman A, Mossabir R, Chaudhry N: **Group psychological intervention for postnatal depression: a nested qualitative study with British South Asian women**. *BMC Womens Health* 2015, **15**(1):109.

For peer review only

49. Edge D: **'We don't see black women here': an exploration of the absence of black Caribbean women from clinical and epidemiological data on perinatal depression in the UK**. *Midwifery* 2008, **24**(4):379–389.

50. McGrath L, Peters S, Wieck A, Wittkowski A: **The process of recovery in women who experienced psychosis following childbirth**. *BMC Psychiatry* 2013, **13**(1):341.

51. Radcliffe P: **Substance-misusing women: Stigma in the maternity setting**. *Br J Midwifery* 2011, **19**(8).

52. Plunkett C, Peters S, Wieck A, Wittkowski A: **A qualitative investigation in the role of the baby in recovery from postpartum psychosis**. *Clin Psychol Psychother* 2017.

53. Almond P, Lathlean J: **Inequity in provision of and access to health visiting postnatal depression services**. *J Adv Nurs* 2011, **67**(11):2350-2362.

54. Cantwell R, Clutton-Brock T, Cooper G, Dawson A, Drife J, Garrod D, Harper A, Hulbert D, Lucas S, McClure J *et al*: **Saving Mothers' Lives: Reviewing maternal deaths to make motherhood safer: 2006-2008. The Eighth Report of the Confidential Enquiries into Maternal Deaths in the United Kingdom**. *BJOG* 2011, **118 Suppl 1**:1-203.

55. Ross-Davie M, Elliott S, Sarkar A, Green L: **A public health role in perinatal mental health: Are midwives ready?** *Br J Midwifery* 2006, **14**(6):330-334.

56. **Stepping Forward to 2020/21: The mental health workforce plan for England July 2017: Mental Health Taskforce strategy** [http://www.rcpsych.ac.uk/pdf/FYFV%20Mental%20health%20workforce%20plan%20for%20England%20FINAL.pdf]

57. **Implementing the Five Year Forward View for mental health** [https://www.england.nhs.uk/wp-content/uploads/2016/07/fyfv-mh.pdf]

For peer review only

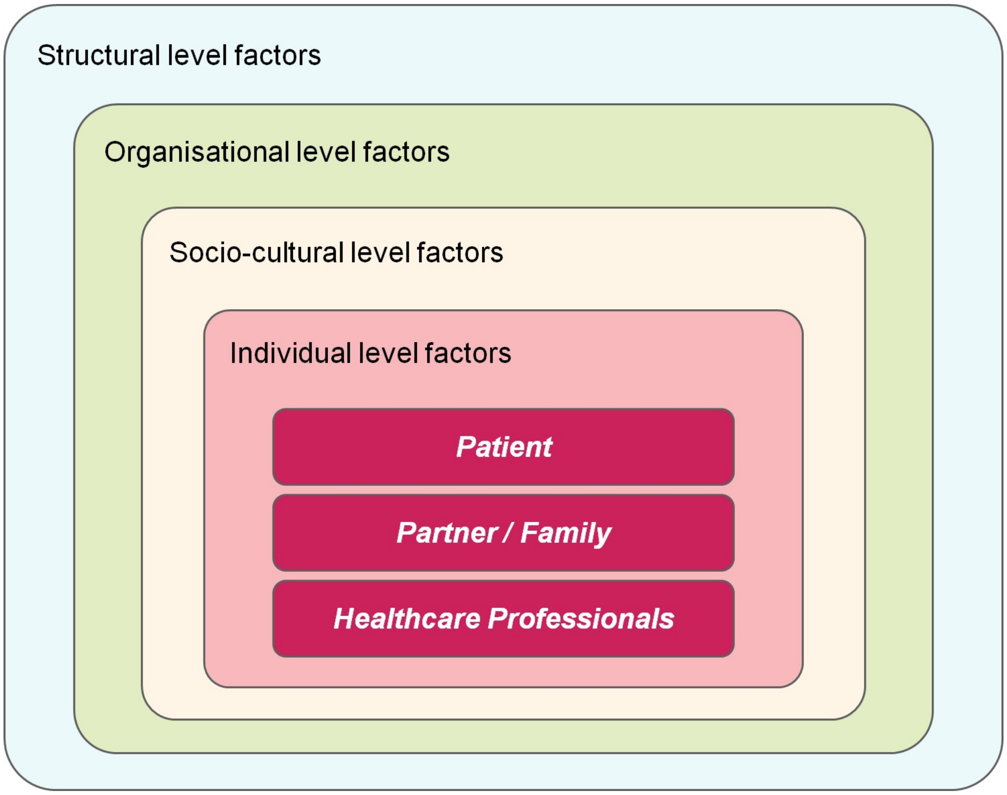

Figure 1: Adapted model showing multi-level conceptual framework for barriers to mental health services in the perinatal period [19,20].

126x99mm (300 x 300 DPI)

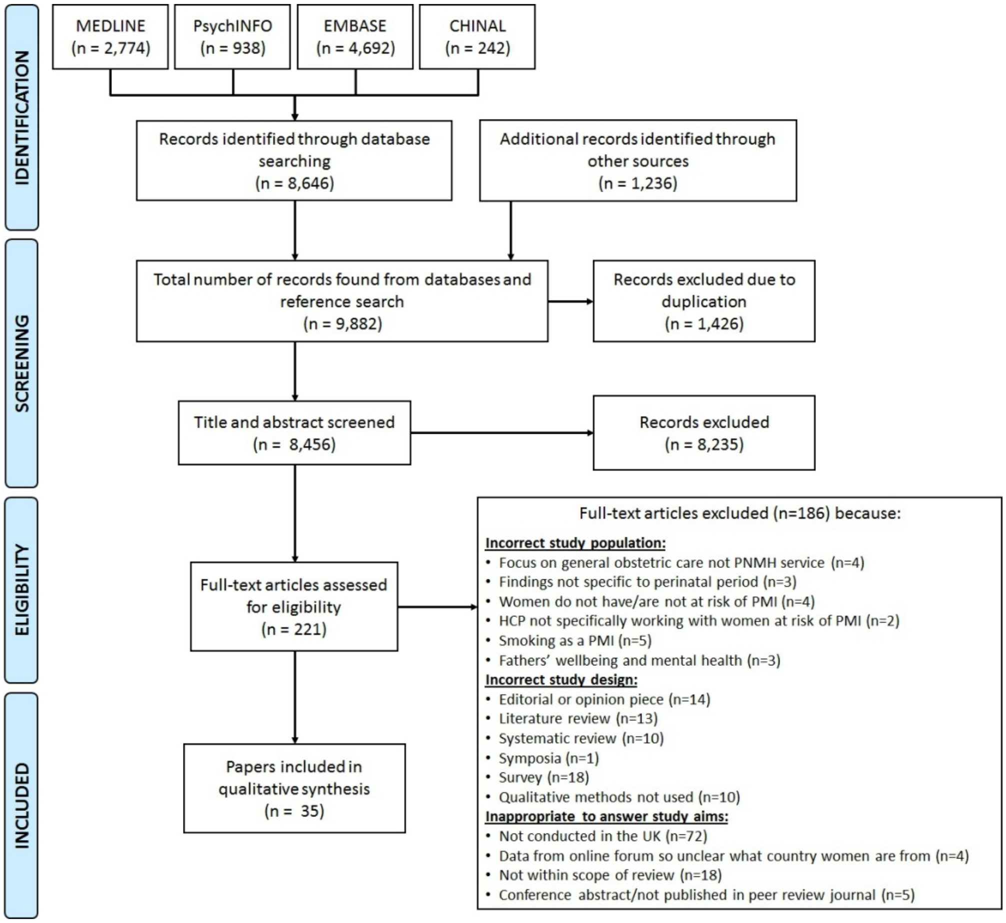

Figure 2: Study Selection

110x101mm (300 x 300 DPI)

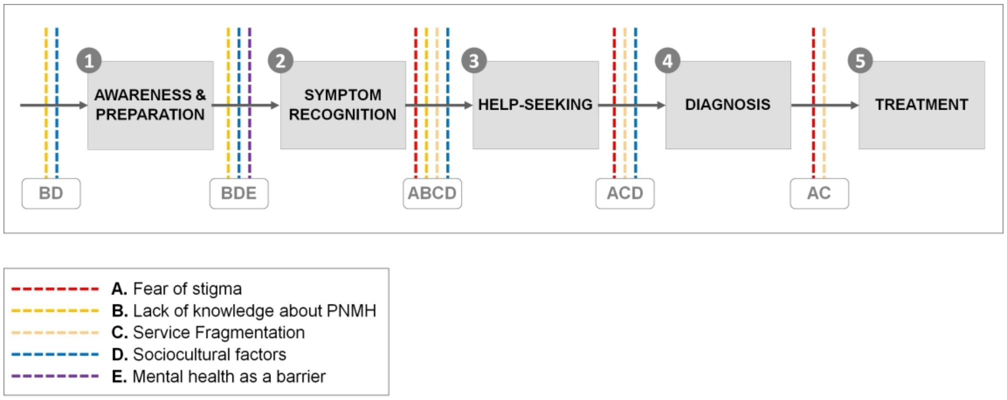

Figure 3: Conceptual model of key barriers in the care pathway to accessing mental health services during the perinatal period

237x93mm (300 x 300 DPI)

Supplementary File 1: Full Search Strategy for Medline via Ovid

1. exp Postnatal Care/
2. exp Perinatal Care/
3. exp Prenatal Care/
4. exp Pregnancy/
5. exp Pregnant Women/
6. exp Parturition/
7. (Perinatal or postnatal or prenatal or pregnan* or antenatal or maternal or postpartum or birth).mp.
8. **OR 1-7**
9. exp Mental Disorders/
10. exp Mental Health/
11. ((mental adj5 health) or (mental adj5 health adj2 problem*) or (mental adj5 illness*) or well-being or psychosis or schizophreni* or anxi* or depressi* or (obsessive compulsive adj5 disorder) or phobi* or (personality adj2 disorder*) or substance abuse).mp.
12. **OR 9-11**
13. exp Health Services Accessibility/ or exp "Delivery of Health Care"/
14. (barrier* or (help adj2 seek*) or (health adj2 seek*) or (support adj2 seek*) or access).mp.
15. **OR 13-14**
16. exp Qualitative Research/
17. exp "Attitude of Health Personnel"/ or exp Health Knowledge, Attitudes, Practice/
18. (Experience* or attitude* or perspective*).mp.
19. (qualitative Research or qualitative or phenomenograph* or grounded theory or ethnograph* or case stud* or interview* or focus group*).mp.
20. **OR 16-19**
21. **8 AND 12 AND 15 AND 20**
22. limit 21 to (english language and yr="2007 -Current")

For peer review only

Supplementary Table 1: Comprehensive summary of data extracted from papers for review

	Author /Year	Study Location	Study Aims	Data collection Method	n =	Study Population	Analytic Strategy and interpretative Theory	Key Emergent Themes Relating to Barriers in Care	CASP Score (/10)
1	Almond (2011)	Southern England	Understanding equity in provision of PND services	Observed discussions between HVs and postnatal women and interviews	46	HVs (n=16) managers (n=6) English women (n=12) Bangladeshi women (n=9), cohesion worker (n=2) and mental health practitioner (n=1).	Thematic content data analysis	-Unacceptability of PND screening<br>-Policy not implemented in practice<br>-Cultural and language barriers	8
2	Baldwin (2009)	Inner London	Assessment of South Asian mothers well-being by Specialist community public health nurses	Interviews	8	SCPHN working with South Asian mother	Content analysis	-Poor support networks<br>-Culturally specific barriers<br>-Domestic and emotional abuse	5
3	Boath (2013)	Stoke-on-Trent	Teenage mothers' experiences of PND and their care	In-depth interviews	15	First-time mothers aged 16-19 who scored >12 on EPDS	Thematic Framework Analysis	-Consequences of disclosure<br>-Poor continuity of care with preferred HCP<br>-Utilisation of family/friends as alternatives for PNMH services<br>-Poor education about perinatal mental health (PNMH) and mental health services (MHS)<br>-Logistical issues	9
4	Boddy (2017)	England	Men's experiences of partner's admission to MBU for first episode PP	In-depth interviews	7	Partners of women receiving inpatient treatment for first episode PP	Interpretive Phenomenological Analysis	-Normalising symptoms<br>- Poor PNMH education<br>-Failures in system to manage emergencies<br>-PNMH as a barrier	10
5	Brown (2009)	Coventry, Warwickshire	Improve understanding of PNMH service access	Focus-group discussions	68	Midwives (n=50) and midwifery students (n=18) working with PMIs	Thematic Framework Analysis	-Unacceptability of PND screening<br>-HCP reluctant to diagnose<br>-Midwives have poor training on PNMH<br>-Normalising symptoms<br>-Policy not implemented in practice<br>-Unclear referral pathways<br>-Delays being seem by specialist<br>-Failures in system to manage emergencies	4
6	Chew-Graham (2008)	Bristol, Manchester London	Determine GPs' and HVs' roles in management of PND	In-depth interviews	23	GPs (n=(n=19) and HVs (n=14) from RESPOND trial	Inductive thematic analysis approach	-HCPs' reluctant to diagnose<br>-Unacceptability of PND screening<br>-Normalising symptoms<br>-Poor education about PNMH and available treatments<br>-Poor continuity of care and communication<br>-Inappropriate HCP attitudes	8

7	Chew-Graham (2009)*	Bristol, Manchester London	GPs, HVs and women's views on disclosure of PND symptoms	In-depth interviews	28	Women from RESPOND trial scoring >11 on EPDS	Inductive thematic analysis approach	-PNMH diagnosis issues<br>-PNMH as a barrier<br>-Stigma and fear of medications<br>-fear of "bad mum" label<br>-poor opinion of HCPs<br>-Unclear roll of HV<br>-"easier not to ask"<br>-inadequate resources	8
8	Coates (2015)	South East England	PNMH symptoms in new mothers and experiences of assessment	interviews at home (n=15) or via telephone (n=2)	17	Women experiencing distress with baby <1year	Inductive thematic analysis approach<br>----------------------<br>*Critical realist approach*	-PNMH diagnosis issues<br>-Unacceptability of PND screening<br>-"it is actually alright to ask for help"<br>-Stigma of PNMH<br>-Inadequate resources<br>-Logistically issues	10
9	Coates (2014)	South East England	Experiences of postpartum emotional distress	interviews at home (n=15) or via telephone (n=2)	17	Women experiencing distress with baby <1year	Interpretive Phenomenological Analysis	-Fear of "bad mum" label<br>-Normalising symptoms<br>-Waiting for help to come<br>-Poor opinion of HCP<br>-Poor support networks	10
10	Cooke (2012)	UK**	Parents' perceptions of barriers to PNMH services	In-depth interviews	7	Parents (>18years) with psychological distress	Thematic Framework Analysis	-Fear of disclosure<br>-Inappropriate HCP attitudes<br>-PNMH as barrier	5
11	Edge (2007)	Northern England	Explain low PND service access among Black Caribbean women	In-depth interviews	12	Black Caribbean women, >18years, scoring >12 on EPDS	Grounded theory approach using constant comparison method	-Poor PNMH awareness<br>-Culturally specific barriers<br>-Fear of "bad mum" label<br>-Fear of medication side effects<br>-Fear of disclosure and stigma<br>-Inappropriate HCPs attitudes	8
12	Edge (2008)	North West England	Explain low PND service access among Black Caribbean women their absence from UK perinatal research	In-depth interviews	12	Black Caribbean women, >18years, scoring >12 on EPDS	Thematic Framework Analysis	-Culturally specific barriers<br>-Inappropriate HCP attitudes<br>-Stigma	10
13	Edge and MacKian (2010)	North West England	Black Caribbean women's accounts of help-seeking for PND	In-depth interviews	12	Black Caribbean women, >18years, scoring >12 on EPDS	Thematic Framework Analysis<br>------------------------<br>Draws on social model of depression and MacKian concepts of 'reflexive Communities'	-Culturally specific barriers<br>-Poor PNMH awareness	9

14	Edge (2010)	Northern England	HCPs views about PNMH care for BME women	In-depth interviews and Focus Group discussions	42	GPs (n=5), midwives (n=22), midwifery managers (n=5), hospital doctors (n=2), HVs (n=5), volunteers (n=3)	Thematic Framework Analysis	-Physical health over PNMH<br>-Policy not implemented<br>-Diagnostic difficulties<br>-Poor interdisciplinary communication<br>-Unclear referral pathways<br>-Lack of resources<br>-Language barriers	9
15	Edge (2011)	Northern England	Black Caribbean women's opinion of PND service access	In-depth interviews	42	Black Caribbean women >18years, with PND or knew someone who had PND	Thematic Framework Analysis	-Previous negative NHS experience<br>-Inadequate resources<br>-Poor support networks<br>-Culture barriers, poor awareness of PNMH	9
16	Evans (2017)	East Midlands	Women's experiences of anxiety in pregnancy and their views on the use of anxiety instruments in antenatal care	Focus group discussions	19	Woman >18 years old who had given birth in last nine months. Women had to have considered themselves as having anxiety during pregnancy	Template analysis	-Not able to share worries with others and feeling isolated<br>-Poor communication<br>-Unclear referral pathways<br>-Assessment tools as a tick box exercise	7
17	Gardner (2014)	Manchester	UK-based West African mothers experiences of PND	In-depth interviews	6	Black West African women scoring >10 on EPDS and using self-help parenting groups	Interpretive Phenomenological Analysis	-Poor PND awareness<br>-Poor support networks<br>-Lack of religious faith<br>-Logistical issues<br>-Stigma	8
18	Glover (2014)	Northern England	Women's experiences of PP and their causal explanations	In-depth interviews	7	Women accessing specialist psychiatric services due episode of PP <10years ago	Inductive thematic analysis<br>------------------------<br>Draws on the stress-vulnerability model	-Normalising symptoms<br>-Poor advice from friends<br>-Poor education about PP<br>-Baby as a barrier<br>-Focus on physical health<br>-Stigma	9
19	Husain (2015)	Manchester Lancashire	Pilot trial of culturally-adapted PHP for British South Asian women with PND	In-depth interviews (Conducted in Urdu, transcribed in English)	17	British South Asian Women with PND from Bangladesh (n=1) Pakistan (n=14) India (n=2). All participants from ROSHNI-2 trial	Thematic Framework Analysis	-Domestic and emotional abuse<br>-Logistical issues<br>-Language and cultural barriers<br>-Lack of anonymity in therapy<br>-Anxiety about treatment	8
20	Jomeen (2013)	Northern England	HVs' experiences of psychological distress and PMI assessment	Focus Group Discussions	5	HVs attending an optional PNMH training day	Thematic Framework Analysis	-Lack of formal PNMH training<br>-Diagnostic issues<br>-Stigma<br>-Issues with interpreters<br>-Poor opinion of HCPs<br>-Issues with referral pathway<br>-Interdisciplinary communication	9

21	Lam (2012)	Manchester	Investigate Chinese women's postnatal experiences	In-depth interviews (conducted in Chinese and English)	8	UK-based, postnatal, Chinese women. EPDS from 2-17***	Grounded theory approach	-Poor information available<br>-Utilisation of family, friends and online forums as an alternative for PNMH services<br>-Language barrier<br>-Fear of family conflict<br>-Conflicting advice from professionals and family<br>-preoccupied with baby	9
22	McGookin (2017)	Manchester	Student Midwives awareness, knowledge and experience of antenatal anxiety	Semi-structured in-depth interviews (19-30 minutes)	7	Student Midwives (year 1-3)	Thematic analysis	-Fear of consequences of disclosure<br>-lack of education and awareness about mental health<br>-poor training<br>-inappropriate staff attitudes<br>-Lack of time and resources	7
23	McGrath (2013)	North West England	Understanding PP recovery	In-depth interviews conducted via phone (n=1) or face-to-face (n=11)	12	Women with any postnatal psychosis according to International classification of Disease- version 10 (ICD-10)	Grounded theory approach<br>--------------------<br>Draws on 'Recovery framework' and 'Biopsychosocial models of illness'	-PNMH as a barrier<br>-Fear of "bad mum" label<br>-Stigma	10
24	Millet (2018)	London	Women's and HCPs experience of access to *Improving Access to Psychological therapies* (IAPT) services during perinatal period	Interviews with service users (n=12) and therapists (n=14)	26	Women referred to mental health services who spoke English and had a baby aged 6-9 months at time of interview. Therapists working for IAPT.	Thematic Analysis	-Normalising symptoms<br>-Fear of "bad mum" label and consequences of disclosure<br>-IAPT not tailored to individual with PNMH conditions<br>-Non baby-friendly environment<br>-Lack of family support	9
25	Nicholls (2007)	Sussex	Experiences of postnatal-PTSD in couples relationship	In-depth interviews	12	Couples where either women (n=3), partner (n=1) or both (n=2) fulfilled DSM-IV criteria for postnatal-PTSD ****	Thematic Framework Analysis	-Difficulty of discussing PNMH<br>-Support networks<br>-Previous negative NHS experience	8
26	Patel (2013)	North West of England	PND illness beliefs	In-depth interviews	11	Women with PND or depression in pregnancy which continued after birth and scored >10 on EPDS	Grounded Theory<br>------------------------<br>Draws on 'Leventhal's self-regulation model of illness beliefs'	-Fear of "bad mum" label<br>-Normalising symptoms and unknown cause of symptoms<br>-Stigma<br>-PNMH as barrier	9
27	Phillips (2015)	UK	Student midwives awareness of PMIs	Focus group discussions (2 groups lasting 1 hour)	9	Student midwives within 6 months of completing degree.	Thematic Framework Analysis	-Poor resources<br>-"easier not to ask"<br>-Inadequate education about PNMH	8

28	Plunkett (2017)	North West England	Role of baby in PP recovery from	In-depth interviews phone (n=7) or face-to-face (n=5)	12	Women who had experienced PP. (checked on a "symptom checklist")	Thematic Framework Analysis<br>----------------------<br>*****	-Baby as motivating recovery factor<br>-Fear of disclosure	10
29	Radcliffe (2011)	Three UK hospital trusts	Provider experiences of services access and stigmatisation for substance-misusing women	In-depth interviews and participant observations	46	Community midwives, specialist midwives, postnatal ward staff, sonographers (n=22). Perinatal women accessing substance-misuse clinics (n=24)	Grounded Theory<br>----------------------<br>Uses a 'social constructionist approach'	-Poor diagnostic tools<br>-Stigma<br>-Logistical issues (eg. lack of diary to keep appointments)	4
30	Rothera (2008)	Trent, Leicestershire, Northampton, Rutland	To highlight issues with PNMH management	In-depth interviews and Focus Group discussions	39	HVs(n=4), Midwife (n=4), GP's (n=3), mental health practitioner (n=2), community psychiatric nurse (n=8), obstetrician (n=5), psychiatrists (n=9), managers (n=4)	Analytic induction and data reduction	-Failure to manage emergencies<br>-Policy not implemented<br>-Poor HCP PHM education and awareness<br>-Interdisciplinary communication	7
31	Rowan (2010)	Southern England	Exploration of policy recommendation implementation	In-depth interviews	8	Managers of maternity services, community midwives, community psychiatric nurses and psychiatrists	Thematic Framework Analysis	-Policy not implemented<br>-Issues with catchment areas<br>-Poor awareness about PNMH treatments	8
32	Slade (2010)	Trent	Women's experiences of HVs identifying and managing PND (part of PoNDER trial).	In-depth interviews	30	Women in PoNDER trial with 6-week PNDS score >18	template approach	-Poor support networks<br>-Fear of disclosure and stigma<br>-Unclear role of HV and poor training<br>-Poor continuity of care<br>-Inadequate resources<br>-Negative experiences of HCP	9
33	Wan (2008)	Manchester Liverpool	Explored providers' perspectives of support needs of mothers with schizophrenia	In-depth interviews	28	Midwives (n=15), obstetric consultant (n=1), psychiatrist (n=1), registered mental health nurses (n=7), manager (n=1) nursery nurse (n=3). Recruited from MBUs (n=9) or antenatal clinics (n=19).	Thematic Framework Analysis	-Fear of consequences<br>-Lack of midwifery training<br>-PNMH as barrier<br>-Interdisciplinary communication<br>-Lack of education about PNMH<br>-Continuity of care<br>-Support networks<br>-Lack of resources	10
34	Wittkowski (2011)	Greater Manchester	South Asian mothers' understanding of PND	In-depth interviews	10	South Asian mothers scoring >12 on EPDS	Grounded Theory approach	-Fear of upsetting others, losing job and stigma<br>-Cultural and language barriers<br>-Racial discrimination<br>-Poor PNMH awareness<br>-Poor social support networks<br>-Interdisciplinary communication	8

35	Wyatt (2015)	England	How women and their significant others make sense of PP experiences	In-depth interviews	7	Women with PP and their carers (partners (n=5), family members (n=1) friends (n=1))	Interpretive Phenomenological Analysis	-Poor social support networks<br>-Fear of "bad mum" label<br>-Poor knowledge of PNMH amongst carers<br>-Feeling guilty for feeling ill	9

Table 4.1 Key:

**Quotes from the Chew-Graham 2008 study population were also included in this paper*

***Paper quotes: "Culturally and socio-economically diverse, urban region of UK"*

****This review will only extract data from participants scoring >10 on EPDS*

*****This review will only extract data from the women within the study diagnosed with birth-related PTSD*

******Paper mentions there is limited theory and pre-existing research on this topic so recognises it may not be possible to map findings onto a formal theory*

For peer review only

Supplementary Table 2: Summary of CASP tool used for quality appraisal of Qualitative Data

Key: ✓= Yes; X= No; ?= Insufficient information to make assessment

Author/Year	Clear Aims	Appropriate methodology	Appropriate research design	Appropriate recruitment strategy	Appropriate data collection methods	Researcher-participant relationship considered	Ethical issues considered	Rigorous data analysis	Clear findings	Value of research	Overall Assessment
Almond (2011)	✓	✓	?	✓	✓	✓	✓	✓	✓	?	Adequate
Baldwin (2009)	✓	?	✓	✓	✓	?	✓	?	X	?	Weak
Boath (2013)	✓	✓	✓	✓	✓	?	✓	✓	✓	✓	Strong
Boddy (2017)	✓	✓	✓	✓	✓	✓	✓	✓	✓	✓	Strong
Brown (2009)	✓	✓	✓	?	?	?	?	?	✓	?	Weak
Chew-Graham (2008)	✓	✓	✓	✓	✓	?	?	✓	✓	✓	Adequate
Chew-Graham (2009)	✓	✓	✓	✓	✓	?	✓	✓	✓	?	Adequate
Coates (2015)	✓	✓	✓	✓	✓	✓	✓	✓	✓	✓	Strong
Coates (2014)	✓	✓	✓	✓	✓	✓	✓	✓	✓	✓	Strong
Cooke (2012)	X	✓	✓	?	?	✓	✓	?	✓	?	Weak
Edge (2007)	✓	✓	✓	✓	✓	?	✓	✓	?	✓	Adequate
Edge (2008)	✓	✓	✓	✓	✓	✓	✓	?	✓	✓	Strong
Edge and MacKian (2010)	✓	✓	✓	✓	✓	?	✓	✓	✓	✓	Strong
Edge (2010)	✓	✓	✓	✓	✓	?	✓	✓	✓	✓	Strong
Edge (2011)	✓	✓	✓	✓	✓	?	✓	✓	✓	✓	Strong
Evans (2017)	✓	✓	✓	✓	✓	?	✓	?	✓	?	Adequate
Gardner (2014)	✓	✓	✓	✓	✓	?	✓	?	✓	✓	Adequate
Glover (2014)	✓	✓	✓	✓	✓	?	✓	✓	✓	✓	Strong
Husain (2015)	✓	✓	✓	✓	✓	✓	✓	?	✓	?	Adequate
Jomeen (2013)	✓	✓	✓	?	✓	✓	✓	✓	✓	✓	Strong
Lam (2012)	✓	✓	✓	✓	✓	✓	?	✓	✓	✓	Strong
McGookin (2017)	✓	✓	✓	✓	✓	?	✓	?	✓	?	Adequate
McGrath (2013)	✓	✓	✓	✓	✓	✓	✓	✓	✓	✓	Strong
Millet (2018)	✓	✓	✓	?	✓	✓	✓	✓	✓	✓	Strong
Nicholls (2007)	✓	✓	✓	✓	?	?	✓	✓	✓	✓	Adequate
Patel (2013)	✓	✓	✓	?	✓	✓	✓	✓	✓	✓	Strong
Phillips (2015)	✓	?	✓	✓	✓	✓	✓	?	✓	✓	Adequate
Plunkett (2017)	✓	✓	✓	✓	✓	✓	✓	✓	✓	✓	Strong
Radcliffe (2011)	X	✓	?	?	✓	?	✓	✓	X	?	Weak
Rothera (2008)	✓	✓	✓	✓	✓	?	?	?	✓	✓	Adequate
Rowan (2010)	✓	✓	✓	✓	✓	?	✓	?	✓	✓	Adequate
Slade (2010)	✓	✓	✓	✓	✓	?	✓	✓	✓	✓	Strong
Wan(2008)	✓	✓	✓	✓	✓	✓	✓	✓	✓	✓	Strong
Wittkowski (2011)	X	✓	✓	?	✓	✓	✓	✓	✓	✓	Adequate
Wyatt (2015)	✓	✓	✓	?	✓	✓	✓	✓	✓	✓	Strong

For peer review only - http://bmjopen.bmj.com/site/about/guidelines.xhtml

PRISMA 2009 Checklist

Section/topic	#	Checklist item	Reported on page #
**TITLE**			
Title	1	Identify the report as a systematic review, meta-analysis, or both.	1
**ABSTRACT**			
Structured summary	2	Provide a structured summary including, as applicable: background; objectives; data sources; study eligibility criteria, participants, and interventions; study appraisal and synthesis methods; results; limitations; conclusions and implications of key findings; systematic review registration number.	2-3
**INTRODUCTION**			
Rationale	3	Describe the rationale for the review in the context of what is already known.	4
Objectives	4	Provide an explicit statement of questions being addressed with reference to participants, interventions, comparisons, outcomes, and study design (PICOS).	4
**METHODS**			
Protocol and registration	5	Indicate if a review protocol exists, if and where it can be accessed (e.g., Web address), and, if available, provide registration information including registration number.	5
Eligibility criteria	6	Specify study characteristics (e.g., PICOS, length of follow-up) and report characteristics (e.g., years considered, language, publication status) used as criteria for eligibility, giving rationale.	5
Information sources	7	Describe all information sources (e.g., databases with dates of coverage, contact with study authors to identify additional studies) in the search and date last searched.	5
Search	8	Present full electronic search strategy for at least one database, including any limits used, such that it could be repeated.	5
Study selection	9	State the process for selecting studies (i.e., screening, eligibility, included in systematic review, and, if applicable, included in the meta-analysis).	5
Data collection process	10	Describe method of data extraction from reports (e.g., piloted forms, independently, in duplicate) and any processes for obtaining and confirming data from investigators.	6
Data items	11	List and define all variables for which data were sought (e.g., PICOS, funding sources) and any assumptions and simplifications made.	6-7
Risk of bias in individual studies	12	Describe methods used for assessing risk of bias of individual studies (including specification of whether this was done at the study or outcome level), and how this information is to be used in any data synthesis.	5-6
Summary measures	13	State the principal summary measures (e.g., risk ratio, difference in means).	6
Synthesis of results	14	Describe the methods of handling data and combining results of studies, if done, including measures of consistency (e.g., I^2) for each meta-analysis.	6

For peer review only - http://bmjopen.bmj.com/site/about/guidelines.xhtml

PRISMA 2009 Checklist

Section/topic	#	Checklist item	Reported on page #
Risk of bias across studies	15	Specify any assessment of risk of bias that may affect the cumulative evidence (e.g., publication bias, selective reporting within studies).	5-6
Additional analyses	16	Describe methods of additional analyses (e.g., sensitivity or subgroup analyses, meta-regression), if done, indicating which were pre-specified.	N/A
**RESULTS**			
Study selection	17	Give numbers of studies screened, assessed for eligibility, and included in the review, with reasons for exclusions at each stage, ideally with a flow diagram.	8
Study characteristics	18	For each study, present characteristics for which data were extracted (e.g., study size, PICOS, follow-up period) and provide the citations.	Table 1 in additional documents
Risk of bias within studies	19	Present data on risk of bias of each study and, if available, any outcome level assessment (see item 12).	7
Results of individual studies	20	For all outcomes considered (benefits or harms), present, for each study: (a) simple summary data for each intervention group (b) effect estimates and confidence intervals, ideally with a forest plot.	7
Synthesis of results	21	Present results of each meta-analysis done, including confidence intervals and measures of consistency.	9-13
Risk of bias across studies	22	Present results of any assessment of risk of bias across studies (see Item 15).	7
Additional analysis	23	Give results of additional analyses, if done (e.g., sensitivity or subgroup analyses, meta-regression [see Item 16]).	N/A
**DISCUSSION**			
Summary of evidence	24	Summarize the main findings including the strength of evidence for each main outcome; consider their relevance to key groups (e.g., healthcare providers, users, and policy makers).	14
Limitations	25	Discuss limitations at study and outcome level (e.g., risk of bias), and at review-level (e.g., incomplete retrieval of identified research, reporting bias).	16
Conclusions	26	Provide a general interpretation of the results in the context of other evidence, and implications for future research.	14-16
**FUNDING**			
Funding	27	Describe sources of funding for the systematic review and other support (e.g., supply of data); role of funders for the systematic review.	17

From: Moher D, Liberati A, Tetzlaff J, Altman DG, The PRISMA Group (2009). Preferred Reporting Items for Systematic Reviews and Meta-Analyses: The PRISMA Statement. PLoS Med 6(7): e1000097. doi:10.1371/journal.pmed1000097

For more information, visit: **www.prisma-statement.org**.

For peer review only - http://bmjopen.bmj.com/site/about/guidelines.xhtml

PRISMA 2009 Checklist

For peer review only
